# Targeted regulation of TAK1 counteracts dystrophinopathy in a DMD mouse model

**DOI:** 10.1172/jci.insight.164768

**Published:** 2023-05-22

**Authors:** Anirban Roy, Tatiana E. Koike, Aniket S. Joshi, Meiricris Tomaz da Silva, Kavya Mathukumalli, Mingfu Wu, Ashok Kumar

**Affiliations:** Department of Pharmacological and Pharmaceutical Sciences, University of Houston College of Pharmacy, Houston, Texas, USA.

**Keywords:** Muscle Biology, Therapeutics, Muscle, Signal transduction, Skeletal muscle

## Abstract

Muscular dystrophies make up a group of genetic neuromuscular disorders that involve severe muscle wasting. TGF-β–activated kinase 1 (TAK1) is an important signaling protein that regulates cell survival, growth, and inflammation. TAK1 has been recently found to promote myofiber growth in the skeletal muscle of adult mice. However, the role of TAK1 in muscle diseases remains poorly understood. In the present study, we have investigated how TAK1 affects the progression of dystrophic phenotype in the mdx mouse model of Duchenne muscular dystrophy (DMD). TAK1 is highly activated in the dystrophic muscle of mdx mice during the peak necrotic phase. While targeted inducible inactivation of TAK1 inhibits myofiber injury in young mdx mice, it results in reduced muscle mass and contractile function. TAK1 inactivation also causes loss of muscle mass in adult mdx mice. By contrast, forced activation of TAK1 through overexpression of TAK1 and TAB1 induces myofiber growth without having any deleterious effect on muscle histopathology. Collectively, our results suggest that TAK1 is a positive regulator of skeletal muscle mass and that targeted regulation of TAK1 can suppress myonecrosis and ameliorate disease progression in DMD.

## Graphical abstract

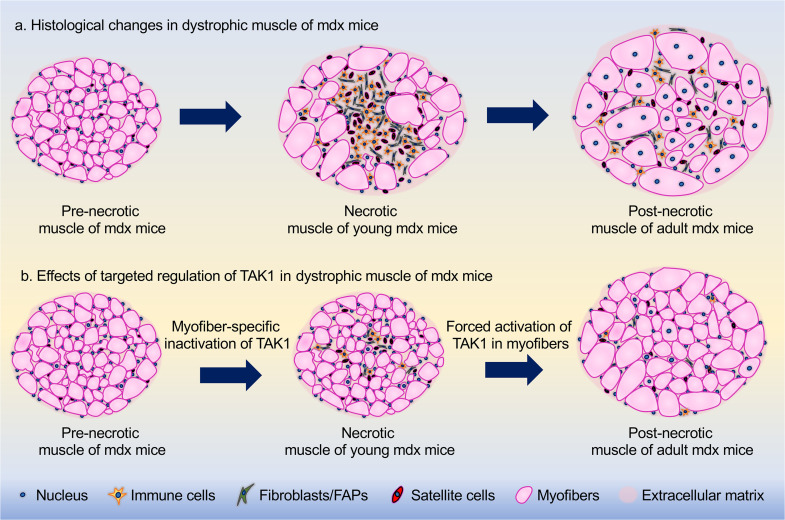

## Introduction

Duchenne muscular dystrophy (DMD) is a severe neuromuscular disease that occurs due to mutations in the DMD gene that encodes the subsarcolemmal protein dystrophin (1). Lack of functional dystrophin protein makes sarcolemma vulnerable to contraction-induced injury that results in degeneration and progressive wasting of nearly all muscles and results in premature death of afflicted individuals (2–4). The DMD pathology onsets with chronic myofiber necrosis and an associated inflammatory response that eventually leads to the replacement of myofibers by fat and fibrotic tissue (5). While emerging new gene-editing technologies, such as CRISPR, to correct defective mutations are prospective therapies for DMD, significant obstacles and safety concerns have thwarted the translation of these approaches to true therapies (1, 6–10). Current standard-of-care for DMD is focused on reducing inflammation with corticosteroids, which modestly reduces disease progression but has serious side effects (11). Accumulating evidence suggests that regulation of immune response, autophagy, and metabolism along with gene correction therapy can be promising approaches to ameliorate disease progression in patients with DMD (12–16).

Destabilization of the dystrophin-glycoprotein complex (DGC) due to dystrophin deficiency inflicts muscle injury and myonecrosis in DMD (17–19). Due to the chronic nature of myofiber degeneration, dystrophic muscle presents a highly complex microenvironment where factors, including immune and other infiltrating cells, inflammatory cytokines, and profibrotic molecules, influence myofiber integrity and regeneration (5, 20). Moreover, aberrant activation of proinflammatory signaling cascades, such as NF-κB and MAPKs, is a common feature in dystrophic muscle (21–28). However, the physiological significance and molecular underpinning that leads to the activation of various signaling pathways in dystrophic muscle remain poorly understood.

TGF-β–activated kinase 1 (TAK1), a member of the MAPK kinase kinase (MAP3K) family, regulates multiple cellular responses, including cell survival, proliferation, differentiation, inflammation, innate immune response, development, and morphogenesis of various organs (29, 30). Numerous cytokines, growth factors, and microbial products initiate cell signaling, which results in the activation of TAK1 signalosome. For activation, TAK1 interacts with adapter protein TAK1 binding protein 1 (TAB1) and either TAB2 or TAB3 and undergoes K63-linked ubiquitination that results in a conformational change leading to TAK1 autophosphorylation within the activation loop (31, 32). Once activated, TAK1 phosphorylates MKK4 and MKK3/6, which leads to the activation of JNK and p38 MAPK, respectively. Another important phosphorylation target of TAK1 is IĸB kinase β (IKKβ), which is responsible for the activation of the canonical NF-κB signaling pathway (31, 33).

We have previously reported that TAK1 is essential for satellite stem cell survival, self-renewal, and myogenic function. Inducible inactivation of TAK1 in satellite cells impairs regeneration of skeletal muscle in adult mice (34). Intriguingly, we also found that TAK1 is critical for the growth and maintenance of skeletal muscle mass. Mice with germline inactivation of *Tak1* in skeletal muscle show perinatal lethality, whereas targeted inducible inactivation of TAK1 causes severe muscle wasting in adult mice, accompanied by activation of proteolytic pathways, repression in the rate of protein synthesis, redox imbalance, and mitochondrial dysfunction (35, 36). More recently, we demonstrated that TAK1 is essential for the integrity of neuromuscular junctions (NMJs) in adult mice. Forced activation of TAK1 through adeno-associated virus–mediated (AAV-mediated) overexpression of TAK1 and TAB1 in skeletal muscle causes myofiber hypertrophy and also attenuates the loss of muscle mass in response to functional denervation (37). A recent study has demonstrated that TAK1 is activated in skeletal muscle of patients with DMD and dystrophic-deficient mdx mice (38). However, the muscle-specific role of TAK1 in disease progression and the mechanisms by which TAK1 regulates dystrophic phenotype in mdx mice remain poorly understood.

In the present study, using genetic mouse models, we have investigated the role of TAK1 in muscle pathogenesis of mdx mice. Our results demonstrate that TAK1 is highly activated during the necrotic phase and that inactivation of TAK1 reduces muscle injury in young mdx mice potentially through inhibiting inflammatory response and necroptosis. However, targeted inactivation of TAK1 strongly inhibits myofiber growth, resulting in reduced muscle mass and strength. In contrast, forced activation of TAK1 in dystrophic muscle of mdx mice through intramuscular injection of AAV6 vectors expressing TAK1 and TAB1 stimulates myofiber growth without having any adverse effect on muscle histopathology. Our experiments suggest that targeted regulation of TAK1 activity can improve muscle mass and contractile function in DMD.

## Results

### TAK1 is activated in dystrophic muscle of mdx mice.

**Figure 1:**
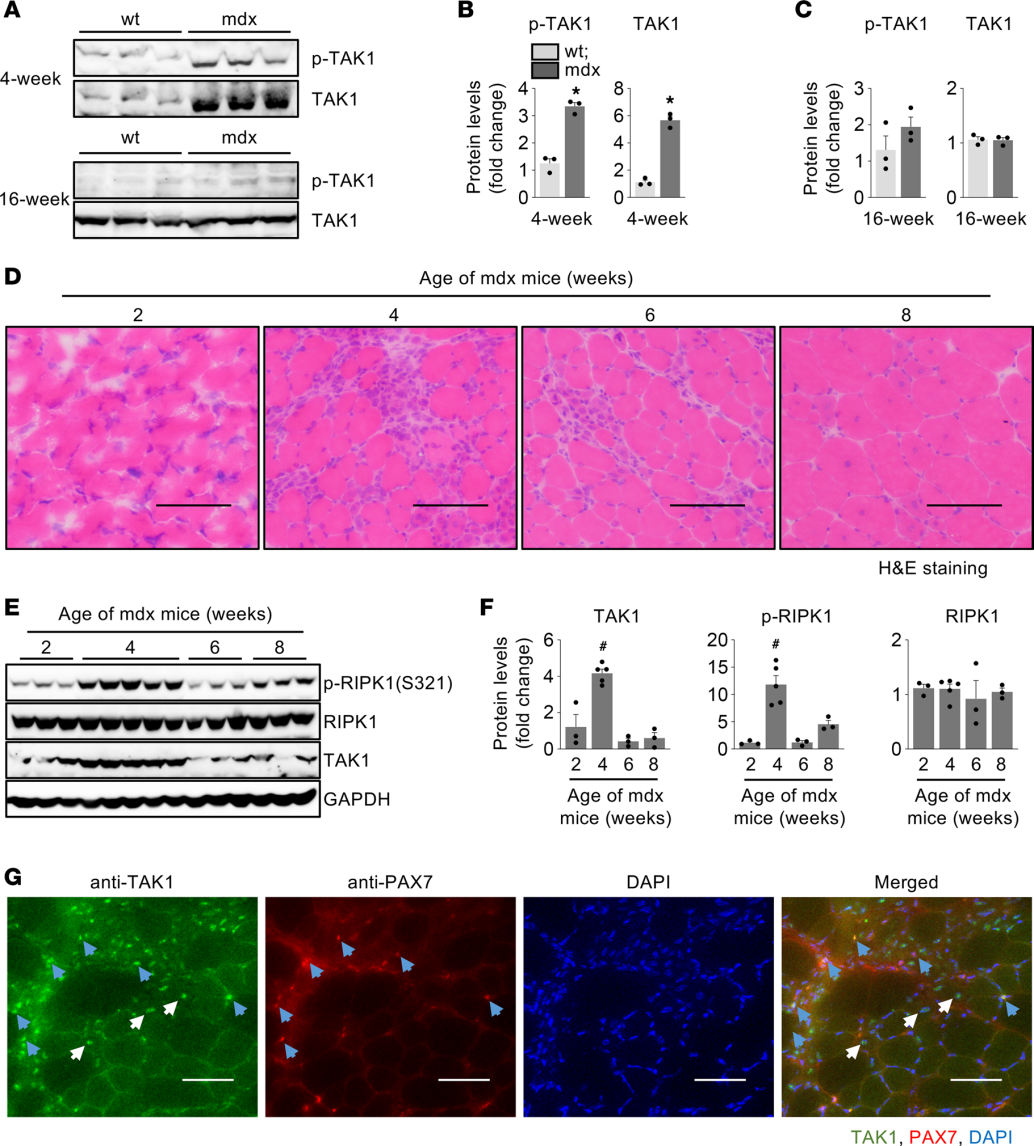
Activation of TAK1 in skeletal muscle of mdx mice. (**A**) Western blot showing protein levels of p-TAK1 and total TAK1 in GA muscle of 4- and 16-week-old WT and mdx mice. (**B** and **C**) Quantification of protein levels of p-TAK1 and total TAK1 in (**B**) 4-week and (**C**) 16-week-old WT and mdx mice. *n* = 3 mice per group. Data represented as mean ± SEM. *P ≤ 0.05, values significantly different from GA muscle of corresponding age-matched WT mice by unpaired Student *t* test. (**D**) Transverse sections of TA muscle from 2-, 4-, 6-, and 8-week-old mdx mice stained with H&E. Scale bar: 100 μm. (**E** and **F**) Western blot and densitometry analysis showing protein levels of TAK1, p-RIPK1 (S321), and total RIPK1 in GA muscle of mdx mice at indicated age. *n* = 3–5. ^#^*P* ≤ 0.05, values significantly different from GA muscle of 2-week-old mdx mice by 1-way ANOVA followed by Tukey’s multiple-comparison test. (**G**) Representative images of TA muscle sections from 4-week-old mdx mice after immunostaining for TAK1 and Pax7 protein. Nuclei was counterstained with DAPI. Scale bar: 50 μm. Blue arrows point to Pax7^+^ satellite cells. White arrows point to myonuclei. *n* = 3–4.

By performing immunoblotting, we first investigated how the levels of TAK1 change with age in the skeletal muscle of dystrophin-deficient mdx mice compared with WT mice. There was a significant increase in the levels of phosphorylated TAK1 (p-TAK1) and total TAK1 protein in the gastrocnemius (GA) muscle of 4-week-old mdx mice compared with age-matched WT mice (Figure 1, A and B). Interestingly, there was no significant difference in the levels of p-TAK1 or total TAK1 in GA muscle of 16-week-old WT and mdx mice (Figure 1, A and C). In the mdx mice, skeletal muscle degeneration starts at the age of 2.5 weeks and severe myonecrosis with inflammation is observed around 3.5–4 weeks. Thereafter, the muscle undergoes a bout of chronic regeneration that continues through adulthood (4, 39). By performing H&E staining, we confirmed significant myonecrosis in tibialis anterior (TA) muscle of 4-week-old mdx mice and appearance of centronucleated myofibers (CNFs) in 6- and 8-week-old mdx mice (Figure 1D). To investigate the temporal expression of TAK1, we measured levels of TAK1 in skeletal muscle of mdx mice of different age groups. Results showed that levels of TAK1 were significantly increased in GA muscle of 4-week-old mdx mice compared with 2-, 6- and 8-week-old mdx mice (Figure 1, E and F). In response to stimulation by TNF-α, TAK1 phosphorylates receptor interacting protein kinase 1 (RIPK1) at Ser321 residue, which leads to cell death through apoptosis or necroptosis (40). Interestingly, myofiber death through necroptosis has been reported to promote muscle degeneration in an mdx model of DMD (41). Consistent with increased activation of TAK1, we observed a significant increase in p-RIPK1 (S321), but not total RIPK1 protein levels, in GA muscle of 4-week-old mdx mice compared with other age groups (Figure 1, E and F). We also examined whether the TAK1 temporal dynamics correlates with the TGF-β ligand dynamics in dystrophic muscle of mdx mice. Results showed that TAK1 was upregulated at time points where latent TGF-β was reduced and active form of TGF-β was increased in TA muscle of mdx mice (Supplemental Figure 1; supplemental material available online with this article; https://doi.org/10.1172/jci.insight.164768DS1). To determine the spatial location of TAK1 during the necrotic phase of mdx pathology, we immunostained transverse sections generated from TA muscle of 4-week-old mdx mice for TAK1 and PAX7 protein. This analysis showed that TAK1 was expressed in regenerating myofibers as well as satellite cells (Figure 1G). Collectively, these results suggest that TAK1 is activated in the skeletal muscle of mdx mice during the peak necrotic phase.

### Inactivation of TAK1 causes loss of skeletal muscle mass and contractile function in mdx mice.

**Figure 2:**
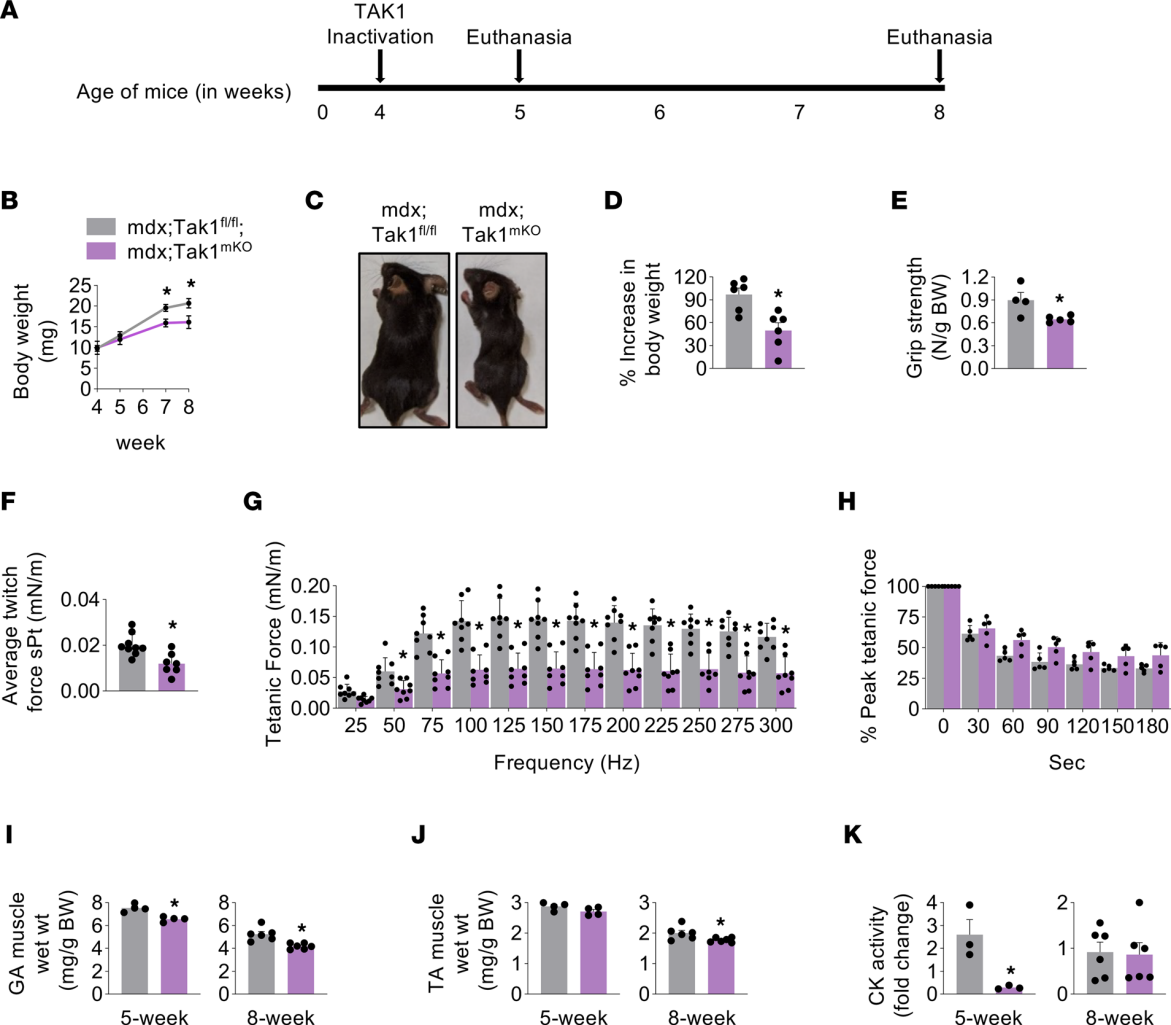
Inactivation of TAK1 in young mdx mice blunts growth and reduces muscle force production. (**A**) Schematic representation showing age of mdx mice, time of TAK1 inactivation, and euthanasia. (**B**) Average body weight of littermate mdx;Tak1^fl/fl^ and mdx;Tak1^mKO^ mice at indicated ages. (**C**) Gross appearance of mdx;Tak1^fl/fl^ and mdx;Tak1^mKO^ mice at the age of 8 weeks. (**D**) Percentage increase in body weight of mdx;Tak1^fl/fl^ and mdx;Tak1^mKO^ mice with age. (**E**) Average 4-paw grip strength per gram of body weight of 8-week-old mdx;Tak1^fl/fl^ and mdx;Tak1^mKO^ mice. *n* = 4–6. (**F**–**H**) Quantification of normalized (**F**) average specific twitch force, (**G**) tetanic forces to stimulation frequency relationship, and (**H**) peak tetanic force over 180 seconds in 8-week-old mdx;Tak1^fl/fl^ and mdx;Tak1^mKO^ mice. *n* = 7–9. (**I**–**K**) Wet weight of (**I**) GA and (**J**) TA muscle and (**K**) fold change in serum levels of CK activity in mdx;Tak1^fl/fl^ and mdx;Tak1^mKO^ mice at 5 and 8 weeks of age. *n* = 3–6. Data represented as mean ± SEM. *P ≤ 0.05, values significantly different from corresponding mdx;Tak1^fl/fl^ mice by unpaired Student *t* test (**B**, **D**, **E**, **F**, **I**, **J**, and **K**) or by 1-way ANOVA (**G** and **H**) followed by Tukey’s multiple-comparison test.

To investigate the muscle role of TAK1 in dystrophic mice, we crossed floxed Tak1 (Tak1^fl/fl^) mice with a skeletal muscle-specific tamoxifen inducible Cre line (HSA-MCM) to generate Tak1^mKO^ mice as described (35, 37). The Tak1^mKO^ mice were then crossed with mdx mice to generate mdx;Tak1^fl/fl^ and mdx;Tak1^mKO^ mice. The second exon of the *Tak1* gene, which encodes the kinase domain, is flanked by loxP sites in these mice. Tamoxifen-mediated inducible expression of Cre recombinase leads to the deletion of the second exon of the *Tak1* gene and significantly reduced the levels of inactivated TAK1 protein (34). For our experiments, 4-week-old littermate mdx;Tak1^fl/fl^ and mdx;Tak1^mKO^ mice were given daily i.p. injections of tamoxifen (75 mg/kg body weight) for 4 consecutive days. The mice were then fed a tamoxifen-containing chow for the entire duration of the experiments. After 1 week or 4 weeks of TAK1 inactivation — i.e., either 5 or 8 weeks of age — the mice were analyzed and euthanized (Figure 2A). TAK1 inactivation in skeletal muscle of mdx mice led to growth retardation (Figure 2B). The body weight of mdx;Tak1^mKO^ mice was significantly less compared with littermate mdx;Tak1^fl/fl^ mice at the age of 7 and 8 weeks (Figure 2B). Moreover, 8-week-old mdx;Tak1^mKO^ mice appeared lean and feeble, besides having significantly reduced accretion of body weight compared with littermate mdx;Tak1^fl/fl^ mice (Figure 2, C and D). In addition, the 4-paw grip strength of mdx;Tak1^mKO^ mice was significantly reduced compared with mdx;Tak1^fl/fl^ mice (Figure 2E). Next, we investigated muscle contractile properties in vivo by measuring the average twitch force, force-frequency relationship, and time to fatigue. Stimulating plantarflexion at different frequencies showed a significant decrease in average twitch force normalized to body weight in mdx;Tak1^mKO^ mice compared with mdx;Tak1^fl/fl^ mice (Figure 2F). Analysis of a force-frequency curve showed significantly less force production at frequencies of 50–300 Hz in mdx;Tak1^mKO^ mice compared with mdx;Tak1^fl/fl^ mice (Figure 2G). However, there was no significant difference in the fatigability between mdx;Tak1^fl/fl^ and mdx;Tak1^mKO^ mice (Figure 2H). We also found reduced GA and TA muscle wet weight in 5- and 8-week-old mdx;Tak1^mKO^ mice compared with corresponding controls (Figure 2, I and J). Serum levels of creatine kinase (CK) activity is an important marker of muscle damage in dystrophic mice (42). Interestingly, we found a significant decrease in CK levels in 5-week-old mdx;Tak1^mKO^ mice but not in 8-week-old mdx;Tak1^mKO^ mice compared with their corresponding controls (Figure 2K). These results suggest that TAK1 is an important regulator of muscle mass and that muscle-specific inactivation of TAK1 blunts skeletal muscle growth and diminishes contractile function in mdx mice.

### Targeted inactivation of TAK1 in young mdx mice reduces muscle injury but restricts myofiber growth.

**Figure 3:**
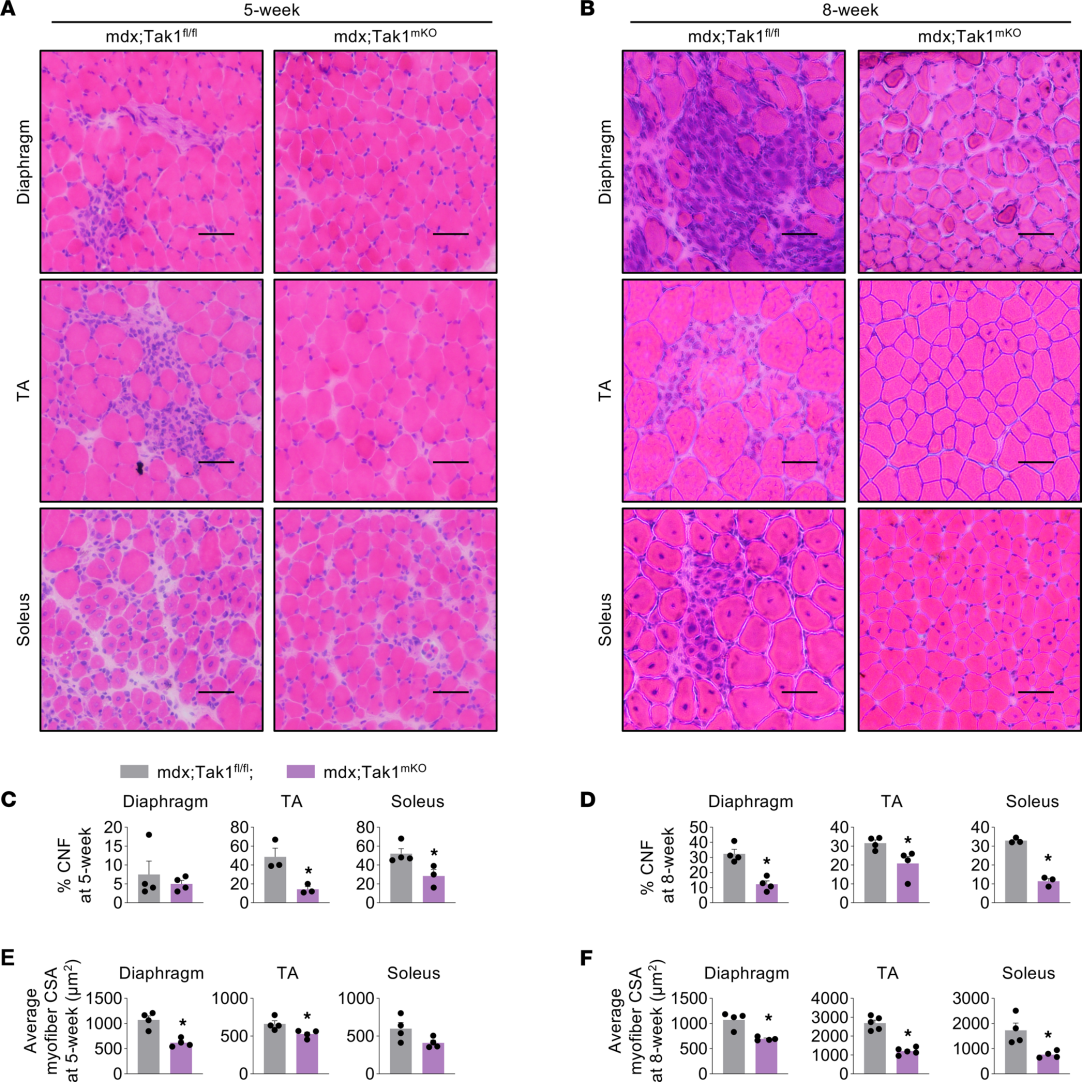
Inactivation of TAK1 improves histopathology but suppresses myofiber growth in young mdx mice. Four-week-old mdx;Tak1^fl/fl^ and mdx;Tak1^mKO^ mice were treated with tamoxifen and analyzed at 5 or 8 weeks of age. (**A** and **B**) Representative photomicrographs of transverse sections of diaphragm, TA, and soleus muscle of (**A**) 5-week-old and (**B**) 8-week-old mdx;Tak1^fl/fl^ and mdx;Tak1^mKO^ mice after staining with H&E. Scale bar: 50 μm. (**C** and **D**) Quantification of percentage of centronucleated fibers (CNF) in diaphragm, TA, and soleus muscle of (**C**) 5-week and (**D**) 8-week-old mdx;Tak1^fl/fl^ and mdx;Tak1^mKO^ mice. (**E** and **F**) Average myofiber cross-section area (CSA) of diaphragm, TA, and soleus muscle of (**E**) 5-week and (**F**) 8-week-old mdx;Tak1^fl/fl^ and mdx;Tak1^mKO^ mice. *n* = 3–5 in each group. Data represented as mean ± SEM. *P ≤ 0.05, values significantly different from corresponding mdx;Tak1^fl/fl^ mice by unpaired Student *t* test.

To understand the effects of inactivation of TAK1 on muscle histopathology, we analyzed transverse sections of diaphragm, TA, and soleus muscle from 5- or 8-week-old mdx;Tak1^fl/fl^ and mdx;Tak1^mKO^ mice after performing H&E staining. In the diaphragm, signs of necrosis and inflammation were sparse at 5 weeks, which were pronounced at 8 weeks in mdx;Tak1^fl/fl^ mice (Figure 3, A and B). In TA muscle, necrosis and cellular infiltration were prevalent at 5 weeks and were less frequent at 8 weeks in mdx;Tak1^fl/fl^ mice (Figure 3, A and B). The soleus muscle sections showed extensive CNFs and cellular infiltration at 5 and 8 weeks of age in mdx;Tak1^fl/fl^ mice (Figure 3, A and B). These histological features were dramatically reduced in 5-week-old and 8-week-old mdx;Tak1^mKO^ mice compared with age-matched mdx;Tak1^fl/fl^ mice (Figure 3, A and B, and Supplemental Figures 2 and 3). Quantitative analysis showed that the proportion of CNFs in the TA and soleus muscle of 5-week-old mdx;Tak1^mKO^ mice was significantly reduced compared with age-matched mdx;Tak1^fl/fl^ mice (Figure 3C). Moreover, diaphragm, TA, and soleus muscle of 8-week-old mdx;Tak1^mKO^ mice also showed a significant reduction in number of CNFs compared with littermate mdx;Tak1^fl/fl^ mice (Figure 3D). Interestingly, the myofibers of mdx;Tak1^mKO^ mice appeared considerably smaller in size compared with mdx;Tak1^fl/fl^ mice (Figure 3, A and B). Indeed, quantitative analysis showed a significant reduction in average myofiber cross-sectional area (CSA) in diaphragm and TA muscle of 5-week-old mdx;Tak1^mKO^ mice compared with littermate mdx;Tak1^fl/fl^ mice (Figure 3E). Similarly, average myofiber CSA in diaphragm, TA, and soleus muscle was also significantly reduced in 8-week-old mdx;Tak1^mKO^ mice compared with littermate controls (Figure 3F). This phenotype was more prominent at 8 weeks of age, where average myofiber CSA in TA and soleus muscle was reduced by more than 50% following TAK1 inactivation.

We also investigated the effect of inactivation of TAK1 on the composition of slow- and fast-type myofibers in skeletal muscle of mdx mice. Results showed that there was a significant increase in the proportion of type IIa myofibers and a significant reduction in type IIb myofibers in TA muscle of 8-week-old mdx;Tak1^mKO^ mice compared with corresponding mdx;Tak1^fl/fl^ mice (Supplemental Figure 4, A and B). Furthermore, a significant decrease in the proportion of type I myofibers and a significant increase in type IIa myofibers were observed in soleus muscle of mdx;Tak1^mKO^ mice compared with mdx;Tak1^fl/fl^ mice (Supplemental Figure 4, A and C). These results suggest that inactivation of TAK1 favors fast-type oxidative myofiber phenotype in skeletal muscle, which may explain similar fatigability in mdx;Tak1^fl/fl^ and mdx;Tak1^mKO^ mice.

### Targeted inactivation of TAK1 does not affect regenerative myogenesis.

**Figure 4:**
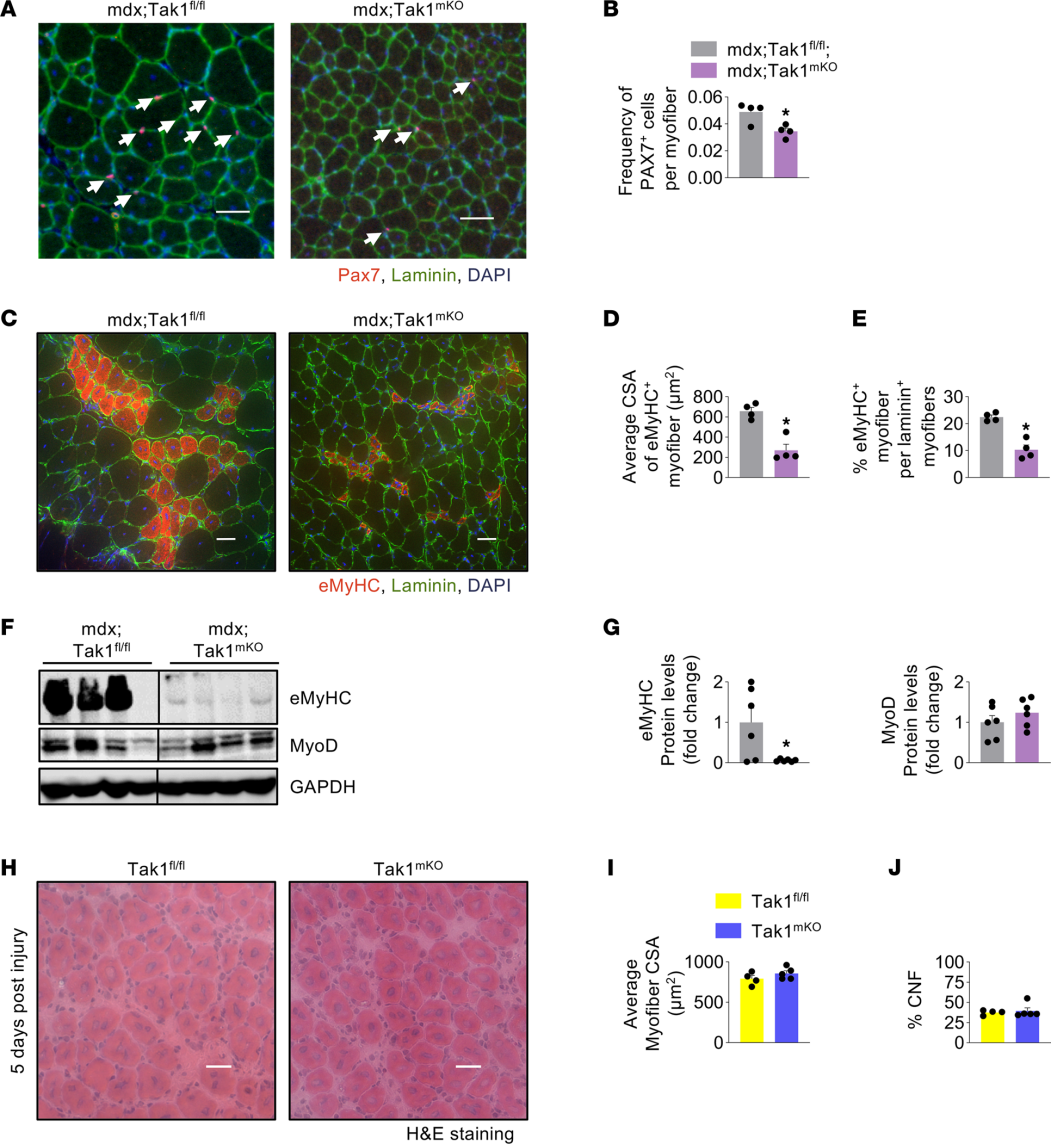
Effect of inactivation of TAK1 on myofiber regeneration in mdx mice. Four-week-old mdx;Tak1^fl/fl^ and mdx;Tak1^mKO^ mice were treated with tamoxifen and analyzed at the age of 8 weeks. Transverse sections of TA muscle were immunostained for Pax7 and Laminin. Nuclei was counterstained with DAPI. (**A** and **B**) Representative photomicrographs and quantitative analysis showing frequency of Pax7^+^ cells in TA muscle of 8-week-old littermate mdx;Tak1^fl/fl^ and mdx;Tak1^mKO^ mice. *n* = 4 per group. Data represented as mean ± SEM. *P ≤ 0.05, values significantly different mdx;Tak1^fl/fl^ mice by Student *t* test. (**C**–**E**) Transverse sections of GA muscle were immunostained for eMyHC and Laminin. Nuclei were counterstained with DAPI. (**C**) Representative photomicrographs and quantitative analysis of (**D**) average myofiber cross-section area of eMyHC^+^ myofibers and (**E**) percentage of eMyHC^+^ myofibers in 8-week-old mdx;Tak1^fl/fl^ and mdx;Tak1^mKO^ mice. *n* = 4 per group. (**F** and **G**) Western blot and densitometry analysis showing protein levels of eMyHC, MyoD, and unrelated protein GAPDH. *n* = 6. Data represented as mean ± SEM. *P ≤ 0.05, values significantly different from mdx;Tak1^fl/fl^ mice by Student *t* test. (**H**–**J**) Following TAK1 inactivation in adult Tak1^fl/fl^ and Tak1^mKO^ mice, TA muscle was injected 100 μL of 10 μM cardiotoxin solution. (**H**) Representative photomicrographs of H&E-stained TA muscle sections and quantification of (**I**) average myofiber cross-section area (CSA) and (**J**) percentage of centronucleated fibers (CNFs). *n* = 4–5 per group. Data represented as mean ± SEM. Western blots for **F**, Figure 5E, and Figure 6E were performed contemporaneously. Black lines on the immunoblots indicate that intervening lanes have been spliced out.

Exhaustion of satellite stem cells and reduced myogenic potential with increasing age is a manifestation of DMD (43). Intriguingly, a few recent studies have suggested that depleting myogenic progenitor cells can ameliorate myopathy severity in animal models of muscular dystrophies (44, 45). Therefore, we sought to investigate whether inducible inactivation of TAK1 affects myofiber regeneration in mdx mice. For this analysis, TAK1 was inactivated in dystrophic muscle of mdx mice at the age of 4 weeks and analyzed at the age of 8 weeks. Immunostaining of TA muscle sections for Pax7 and laminin protein showed a significant reduction in the abundance of Pax7^+^ satellite cells in mdx;Tak1^mKO^ mice compared with mdx;Tak1^fl/fl^ mice (Figure 4, A and B). Embryonic myosin heavy chain (eMyHC) isoform is expressed by newly formed myofibers in regenerating skeletal muscle. Interestingly, the number and size of eMyHC-expressing myofibers were significantly reduced in dystrophic muscle of mdx;Tak1^mKO^ mice compared with mdx;Tak1^fl/fl^ mice (Figure 4, C–E). Western blot analysis showed that there was a significant reduction in the protein levels of eMyHC (Myh3) in GA muscle of mdx;Tak1^mKO^ mice compared with mdx;Tak1^fl/fl^ mice. By contrast, levels of MyoD protein remained comparable between mdx;Tak1^fl/fl^ and Tak1^mKO^ mice (Figure 4, F and G). Fewer satellite cells and diminished levels of eMyHC protein can be indicative of reduced muscle injury or a defect in myofiber regeneration. To delineate between these 2 possibilities, we studied the effect of myofiber-specific inactivation of TAK1 on muscle regeneration in WT mice. TA muscle of adult Tak1^fl/fl^ and Tak1^mKO^ mice was injured by giving intramuscular injections of cardiotoxin, and muscle regeneration was studied 5 days later by morphometric assays. There was no apparent difference in TA muscle regeneration between Tak1^fl/fl^ and Tak1^mKO^ mice (Figure 4H). Quantitative analysis showed that the average myofiber CSA and the proportion of CNFs remained comparable in regenerating TA muscle of Tak1^fl/fl^ and Tak1^mKO^ mice (Figure 4, I and J). These results further suggest that myofiber-specific inactivation of TAK1 in mdx mice mitigates muscle injury without having any effect on muscle regeneration.

### TAK1 induces the phosphorylation of RIPK1 in skeletal muscle of mdx mice.

**Figure 5:**
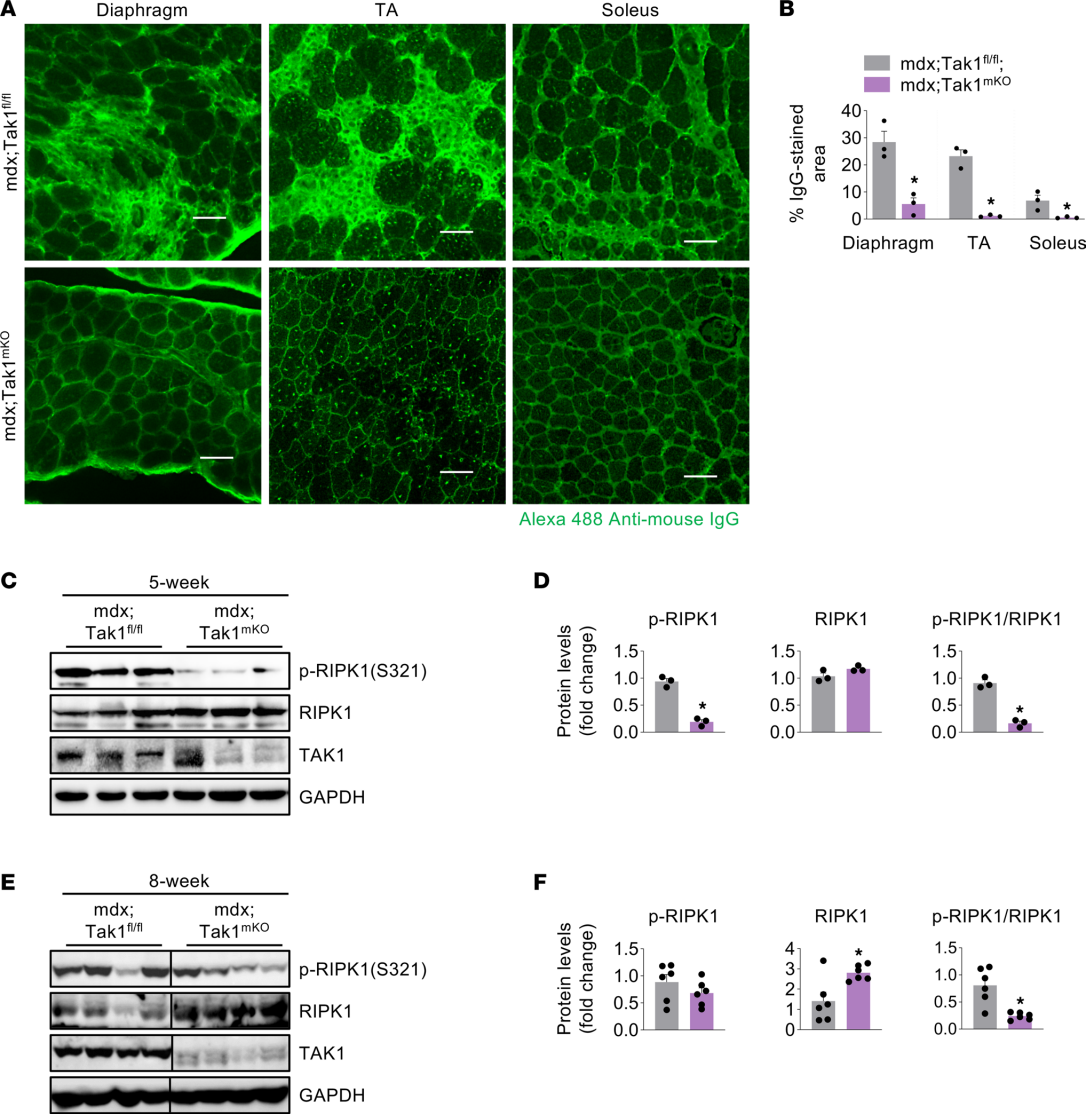
TAK1 contributes to RIPK1 phosphorylation in dystrophic muscle. (**A**) Representative photomicrographs of transverse sections of diaphragm, TA, and soleus muscle of 8-week-old mdx;Tak1^fl/fl^ and mdx;Tak1^mKO^ mice stained with anti–mouse IgG. Scale bar: 50 μm. (**B**) Percentage of IgG-stained area in diaphragm, TA, and soleus muscle sections of 8-week-old mdx;Tak1^fl/fl^ and mdx;Tak1^mKO^ mice. (**C**) Western blot showing protein levels of p-RIPK1 (S321), total RIPK1, and TAK1. (**D**) Densitometry analysis showing fold change of p-RIPK and RIPK1 and ratio of p-RIPK1/total RIPK1 in GA muscle of 5-week-old mdx;Tak1^fl/fl^ and mdx;Tak1^mKO^ mice. *n* = 3 per group. (**E** and **F**) Western blot showing protein levels of p-RIPK1 (S321), total RIPK1, and TAK1, and densitometry analysis showing fold change of p-RIPK and RIPK1 and ratio of p-RIPK1/total RIPK1 in GA muscle of 8-week-old mdx;Tak1^fl/fl^ and mdx;Tak1^mKO^ mice. *n* = 6. Data represented as mean ± SEM. *P ≤ 0.05, values significantly different from mdx;Tak1^fl/fl^ mice by unpaired Student *t* test. Western blots for **E**, Figure 4F, and Figure 6E were performed contemporaneously. Black lines on the immunoblots indicate that intervening lanes have been spliced out.

Mutations in the dystrophin gene destabilizes the DGC that leads to severe myofiber necrosis in skeletal muscle (46). To investigate the extent of necrosis, transverse sections of diaphragm, TA, and soleus muscle from 8-week-old mdx;Tak1^fl/fl^ and mdx;Tak1^mKO^ mice were stained with anti–mouse IgG. There was a significant reduction in IgG-stained areas in skeletal muscle of mdx;Tak1^mKO^ mice compared with mdx;Tak1^fl/fl^ mice (Figure 5, A and B). A recent study has shown that programmed necrosis or necroptosis is pronouncedly activated in the mdx model of DMD and is responsible for RIPK1-dependent myofiber death and inflammation (41). Sustained phosphorylation at the intermediate domain of RIPK1 (S321) by TAK1 has been demonstrated to trigger necroptosis (40). To investigate whether TAK1 regulates RIPK1 in skeletal muscle of mdx mice, we inactivated TAK1 in skeletal muscle of 4-week-old mdx mice. Results show a significant reduction in p-RIPK1 (S321) levels and in p-RIPK1/total RIPK1 ratio in GA muscle of mdx;Tak1^mKO^ mice compared with mdx;Tak1^fl/fl^ mice at 5 weeks of age (Figure 5, C and D). At 8 weeks of age, mdx;Tak1^mKO^ mice showed a significant reduction in the p-RIPK1/total RIPK1 ratio but showed an elevated total RIPK1 level when compared with mdx;Tak1^fl/fl^ mice (Figure 5, E and F). In response to myofiber injury, inflammatory immune cells, such as macrophages, infiltrate into dystrophic muscle (47, 48). Immunostaining for F4/80 antigen, a marker for murine macrophages, showed a significant decrease in the number of F4/80^+^ cells in diaphragm, TA, and soleus muscle of 8-week-old mdx;Tak1^mKO^ mice compared with littermate mdx;Tak1^fl/fl^ mice (Supplemental Figure 5, A and B). We also found a significant reduction in the gene expression of macrophage cell–surface antigens F4/80, CD11c, and CD206 in the GA muscle of 8-week-old mdx;Tak1^mKO^ mice compared with littermate mdx;Tak1^fl/fl^ mice (Supplemental Figure 5C). These results suggest that inactivation of TAK1 attenuates myonecrosis and limits inflammation in dystrophic muscle.

### TAK1 regulates proteolytic systems in dystrophic muscle.

**Figure 6:**
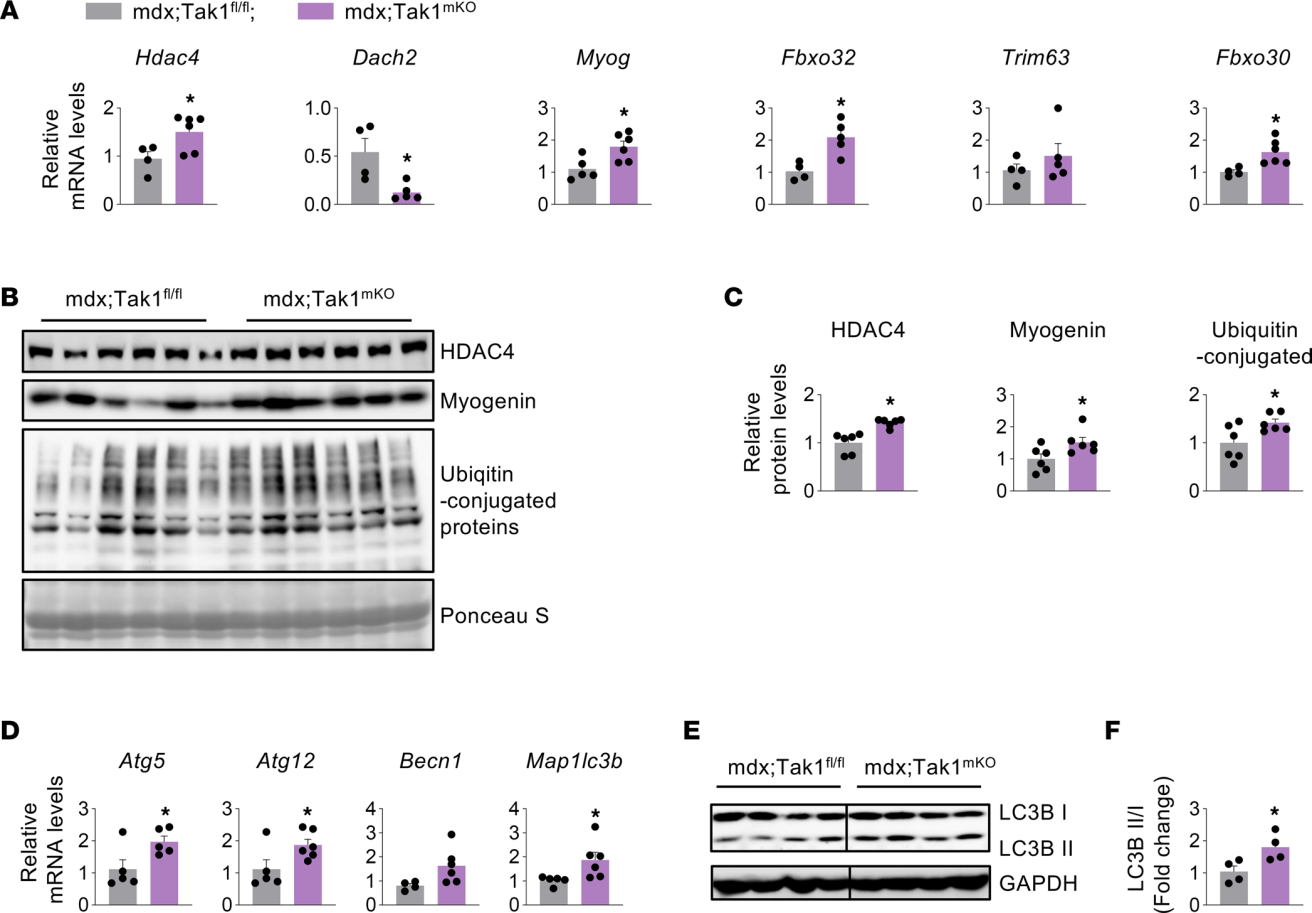
Inactivation of TAK1 stimulates proteolytic systems in dystrophic muscle. Four-week-old mdx;Tak1^fl/fl^ and mdx;Tak1^mKO^ mice were treated with tamoxifen and analyzed at the age of 8 weeks. (**A**) qPCR assay showing relative mRNA levels of *Hdac4*, *Dach2*, *Myog* (Myogenin), *Fbxo32* (MAFbx*)*, *Trim63* (MuRF1), and *Fbxo30* (MUSA1) in 8-week-old mdx;Tak1^fl/fl^ and mdx;Tak1^mKO^ mice. *n* = 4–6. (**B** and **C**) Western blot and densitometry analysis showing protein levels of HDAC4, myogenin, and ubiquitin-conjugated proteins in 8-week-old mdx;Tak1^fl/fl^ and mdx;Tak1^mKO^ mice. *n* = 6. Ponceau S staining showing equal loading of protein. (**D**) Relative mRNA levels of *Atg5, Atg12*, *Becn1* (Beclin1), and *Map1lc3b* (Lc3b) in GA muscle of 8-week-old mdx;Tak1^fl/fl^ and mdx;Tak1^mKO^ mice. *n* = 4–6. (**E** and **F**) Western blot and densitometry analysis showing the ratio of LC3BII to LC3BI in GA muscle of 8-week-old mdx;Tak1^fl/fl^ and mdx;Tak1^mKO^ mice. *n* = 4. Data represented as mean ± SEM. *P ≤ 0.05, values significantly different from mdx;Tak1^fl/fl^ mice by unpaired Student *t* test. Western blots for **E**, Figure 4F, and Figure 5E were performed contemporaneously. Black lines on the immunoblots indicate that intervening lanes have been spliced out.

Under catabolic conditions, muscle proteolysis is primarily mediated by the ubiquitin-proteasome system (UPS) and autophagy (49). Targeted inactivation of TAK1 leads to muscle atrophy, which is accompanied by increased expression of the components of UPS and autophagy (35, 37). The HDAC4-Dach2-myogenin axis has been found to be one of the most important inducers of the gene expression of E3 ubiquitin ligases, MAFbx and MuRF1, in skeletal muscle following denervation. This pathway is also highly activated in skeletal muscle of WT mice following TAK1 inactivation (37). We next sought to investigate whether inactivation of TAK1 affects the markers of UPS and autophagy in dystrophic muscle of mdx mice. Results show that the mRNA levels of *Hdac4*, Myogenin (*Myog*), MAFbx (*Fbxo32*), and MUSA1 (*Fbxo30*) were significantly increased, whereas mRNA levels of *Dach2* were significantly reduced, in GA muscle of 8-week-old mdx;Tak1^mKO^ mice compared with littermate mdx;Tak1^fl/fl^ mice (Figure 6A). Western blot analysis also showed a significant increase in the levels of HDAC4, myogenin, and ubiquitin-conjugated proteins in GA muscle of 8-week-old mdx;Tak1^mKO^ mice compared with age-matched mdx;Tak1^fl/fl^ mice (Figure 6, B and C). Furthermore, mRNA levels of autophagy-related molecules: *Atg5*, *Atg12*, and LC3B (*Map1lc3b*) ratio of LC3BII/LC3BI protein were significantly increased in dystrophic muscle of mdx;Tak1^mKO^ mice compared with mdx;Tak1^fl/fl^ mice (Figure 6, D–F).

We have recently reported that inactivation of TAK1 disrupts TGF-β–induced Smad2/3 and bone morphogenic protein–induced (BMP-induced) Smad1/5/8 signaling in skeletal muscle of WT mice (37). Interestingly, our analysis showed that there was no difference in activation of various markers of these pathways in dystrophic muscle of mdx;Tak1^fl/fl^ and mdx;Tak1^mKO^ mice. The phosphorylated and total levels of Smad2 and Smad1/5/8 proteins remained comparable in skeletal muscle of mdx;Tak1^mKO^ mice and mdx;Tak1^fl/fl^ mice (Supplemental Figure 6, A and B). Moreover, quantitative PCR (qPCR) analysis showed that, even though transcript levels of *Bmr1b* and *Acvr1b* were significantly increased, there was no significant difference in the transcript levels *Bmpr1a*, *Bmpr2*, *Acvr1c*, *Tgfbr1*, *Acvr1a*, *Bmp7*, *Bmp8a*, *Bmp8b*, *Bmp13*, *Tgfb1*, *Tgfb2*, and *Tgfb3* in GA muscle of mdx;Tak1^mKO^ mice compared with mdx;Tak1^fl/fl^ mice (Supplemental Figure 6C). These results suggest that inactivation of TAK1 stimulates the proteolytic system without having any significant influence on TGF-β– or BMP-Smad signaling in dystrophic muscle of mdx mice.

### Targeted inactivation of TAK1 in postnecrotic mdx mice causes muscle wasting.

**Figure 7:**
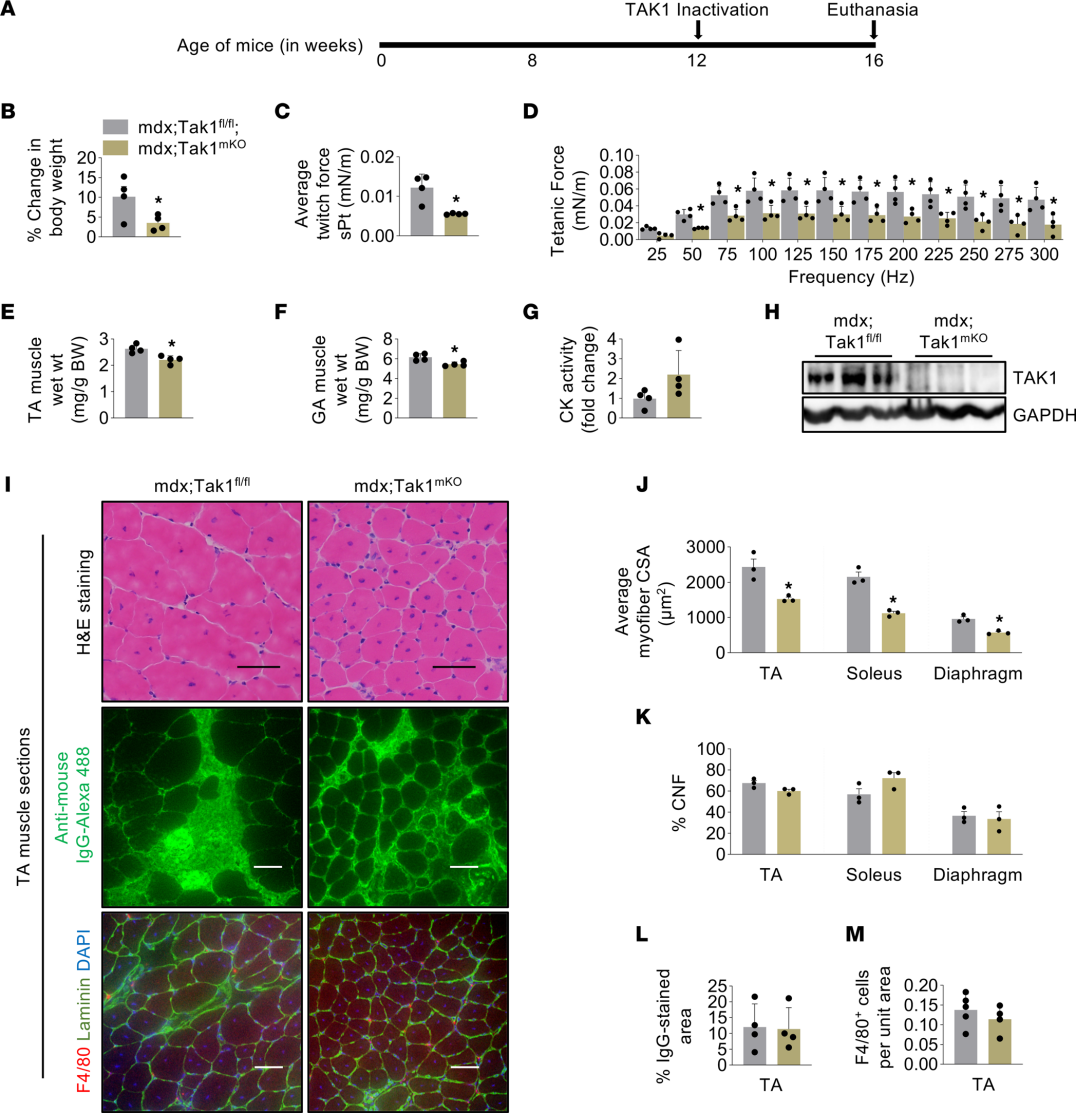
Targeted ablation of TAK1 in adult mdx mice causes muscle wasting without affecting histopathology. (**A**) Schematic representation showing the age of mice and time of treatment with tamoxifen and euthanasia. (**B**) Percentage change in body weight from initial weight of mdx;Tak1^fl/fl^ and mdx;Tak1^mKO^ mice. (**C** and **D**) Normalized average specific twitch force and tetanic force with increasing stimulation frequency in 16-week-old mdx;Tak1^fl/fl^ and mdx;Tak1^mKO^ mice. (**E**–**G**) Wet weight of TA and GA muscle normalized with body weight serum levels of CK in 16-week-old mdx;Tak1^fl/fl^ and mdx;Tak1^mKO^ mice. *n* = 4. (**H**) Western blot showing protein levels of TAK1 in GA muscle of mdx;Tak1^fl/fl^ and mdx;Tak1^mKO^ mice. (**I**) Representative photomicrographs of TA muscle sections stained with H&E or anti–mouse IgG or coimmunostained for F4/80, Laminin, and DAPI. (**J** and **K**) Average myofiber cross-section area and percentage of centronucleated fibers (CNFs) in diaphragm, TA, and soleus muscle of mdx;Tak1^fl/fl^ and mdx;Tak1^mKO^ mice. *n* = 3 per group. (**L** and **M**) Quantitative analysis of (**L**) percentage of IgG-stained area and (**M**) F4/80^+^ cells per unit area in TA muscle of 16-week-old mdx;Tak1^fl/fl^ and mdx;Tak1^mKO^ mice. *n* = 4 per group. Data represented as mean ± SEM. *P ≤ 0.05, values significantly different from GA muscle of 16-week-old mdx;Tak1^fl/fl^ mice by unpaired Student *t* test.

We have previously reported that TAK1 is essential for maintaining skeletal muscle mass in adult WT mice (35). However, the role of TAK1 in the regulation of skeletal muscle mass at the postnecrotic phase in mdx mice remains unknown. Therefore, we inactivated TAK1 in 12-week-old adult littermate mdx;Tak1^fl/fl^ and mdx;Tak1^mKO^ mice and performed the morphometric and biochemical analysis at the age of 16 weeks (Figure 7A). There was a significant reduction in body weight of mdx;Tak1^mKO^ mice compared with littermate mdx;Tak1^fl/fl^ mice (Figure 7B). There was also a significant reduction in specific twitch force normalized to body weight (Figure 7C) and tetanic force production with stimulation ranging from 50 to 300 Hz in mdx;Tak1^mKO^ mice compared with mdx;Tak1^fl/fl^ mice (Figure 7D). Furthermore, wet weight of TA and GA muscle was significantly reduced in mdx;Tak1^mKO^ mice compared with mdx;Tak1^fl/fl^ mice (Figure 7, E and F). However, serum levels of CK remained comparable between mdx;Tak1^fl/fl^ and mdx;Tak1^mKO^ mice (Figure 7G). By performing Western blot, we confirmed a drastic reduction in the levels of TAK1 protein in the skeletal muscle of mdx;Tak1^mKO^ mice compared with corresponding mdx;Tak1^fl/fl^ mice (Figure 7H).

To investigate the effect of inactivation of TAK1 on muscle histopathology in postnecrotic mdx mice, transverse sections of diaphragm, TA, and soleus muscle were analyzed by performing H&E staining. Results showed a significant reduction in average myofiber CSA in skeletal muscle of mdx;Tak1^mKO^ mice compared with corresponding mdx;Tak1^fl/fl^ mice (Figure 7, I and J, and Supplemental Figure 7A). However, the proportion of CNFs remained comparable between mdx;Tak1^fl/fl^ and mdx;Tak1^mKO^ mice (Figure 7, I and K, and Supplemental Figure 7A). Additionally, we did not find any difference in area under necrosis between mdx;Tak1^mKO^ and mdx;Tak1^fl/fl^ mice analyzed by staining of muscle sections with anti–mouse IgG (Figure 7, I and L, and Supplemental Figure 7B). To investigate whether muscle-specific inactivation of TAK1 affects macrophage infiltration in dystrophic muscle of adult mdx mice, we immunostained muscle sections for F4/80 antigen. This analysis showed that the number of F4/80^+^ cells was comparable between mdx;Tak1^fl/fl^ and mdx;Tak1^mKO^ mice (Figure 7, I and M, and Supplemental Figure 7C).

We also performed Sirius red staining to evaluate the collagen levels. However, there was no apparent difference in collagen deposition in diaphragm of 16-week-old mdx;Tak1^fl/fl^ and mdx;Tak1^mKO^ mice (Supplemental Figure 7D). Moreover, mRNA levels of Col1a1, Col2a1, Col3a1, and Col4a1 remained comparable in GA muscle of mdx;Tak1^fl/fl^ and mdx;Tak1^mKO^ mice (Supplemental Figure 7E). Collectively, these results suggest that inactivation of TAK1 in adult mdx mice leads to loss of skeletal muscle mass without affecting muscle histopathology.

### Forced activation of TAK1 augments myofiber growth in adult mdx mice.

**Figure 8:**
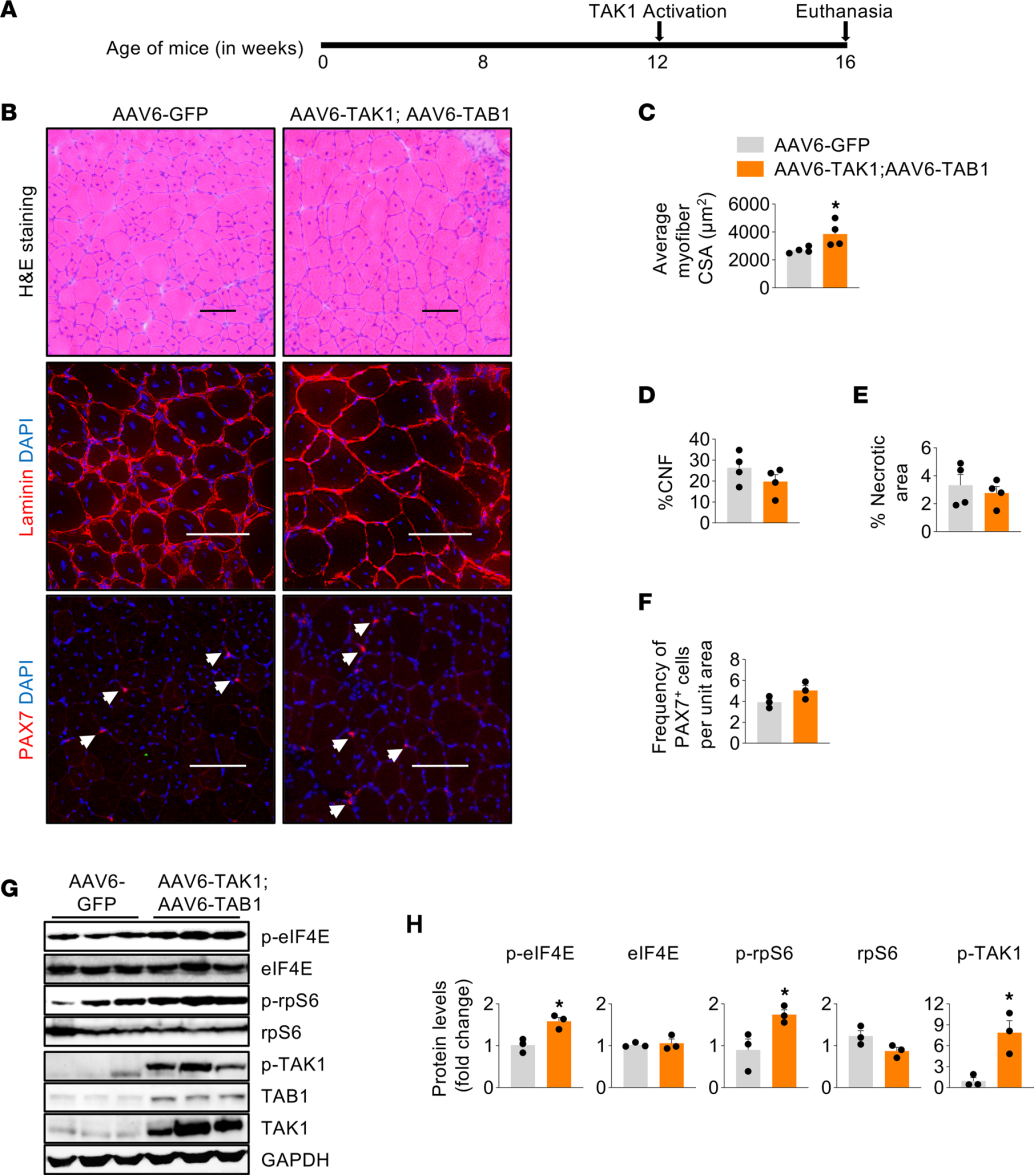
TAK1 activation promotes myofiber growth in adult mdx mice. (**A**) Schematic representation showing the age of mdx mice, time of AAVs injection, and euthanasia. (**B**) Representative photomicrographs of transverse muscle sections after performing H&E staining or immunostaining for laminin or Pax7 protein. Nuclei were counterstained with DAPI. Scale bar: 100 μm. (**C**–**E**) Average myofiber cross-section area, percentage of centronucleated fibers (CNFs), and percentage necrotic area in TA muscle of mdx mice injected with AAV6-GFP or a combination of AAV6-TAK1 and AAV6-TAB1. (**F**) Frequency of Pax7^+^ cells per unit area in TA muscle of mdx mice injected with AAV6-GFP or a combination of AAV6-TAK1 and AAV6-TAB1. *n* = 4 per group. (**G** and **H**) Western blot and densitometry analysis showing protein levels of p-eIF4E, eIF4E, p-rpS6, rpS6, p-TAK1, TAB1, TAK1, and unrelated protein GAPDH in TA muscle of mdx mice injected with AAV6-GFP or coinjected with AAV6-TAK1 and AAV6-TAB1. *n* = 3 per group. Data represented as mean ± SEM. *P ≤ 0.05, values significantly different from TA muscle of mdx mice injected with AAV6-GFP.

We next sought to determine the effect of forced activation of TAK1 in mdx mice through intramuscular injections of AAV6 vectors expressing TAK1 or TAB1 using cytomegalovirus (CMV) promoter (37). Our initial analysis showed that intramuscular injection of about 2 × 10^10^ vgs of AAV6-GFP vectors in TA muscle of mdx mice results in GFP expression in almost all myofibers at day 28 after injection (Supplemental Figure 8A). Therefore, we transduced TA muscle of 12-week-old mdx mice by injecting AAV6-TAK1 (1.25 × 10^10^ vg) and TAB1 (1.25 × 10^10^ vg). The contralateral TA muscle was injected with AAV6-GFP (2.5 × 10^10^ vg) vector and served as a control. After 28 days, the mice were euthanized and the TA muscle was analyzed by morphometric and biochemical assays (Figure 8A). H&E staining did not show any overt histological changes in TA muscle coinjected with AAV6-TAK1 and AAV6-TAB1 or with AAV6-GFP alone. The necrotic areas remained filled with CNFs, a typical feature in skeletal muscle of adult mdx mice (Figure 8B). Sirius red staining also did not show any noticeable difference in collagen deposition in TA muscle coinjected with AAV6-TAK1 and AAV6-TAB1 or AAV6-GFP alone (Supplemental Figure 8B). Anti-laminin staining followed by quantitative analysis showed that average myofiber CSA was significantly higher in TA muscle injected with AAV6-TAK1 and AAV6-TAB1 compared with contralateral TA muscle injected with AAV6-GFP alone (Figure 8, B and C). However, area under necrosis and the proportion of CNFs remained comparable between TA muscle of mdx mice injected with AAV6-GFP alone or with a combination of AAV6-TAK1 and AAV6-TAB1 (Figure 8, B, D, and E). We also investigated the effect of TAK1 and TAB1 overexpression on the number of satellite cells in dystrophic muscle of mdx mice. Results show that the abundance of Pax7^+^ satellite cells was comparable in TA muscle of mdx mice co-overexpressing TAK1 and TAB1 or GFP alone (Figure 8, B and F). To understand the mechanisms of myofiber hypertrophy in mdx mice following activation of TAK1, we measured levels of a few proteins known to regulate skeletal muscle growth. A significant increase in the levels of p-rpS6 and p-eIF4E, along with p-TAK1, total TAB1, and total TAK1, was evidenced in TA muscle overexpressing TAK1 and TAB1 compared with GFP alone (Figure 8, G and H). These results suggest that, similar to WT mice (37), forced activation of TAK1 through intramuscular injection of AAV6-TAK1 and AAV6-TAB1 causes myofiber growth potentially through stimulating protein translation in mdx mice.

### Targeted regulation of TAK1 improves muscle histopathology.

**Figure 9:**
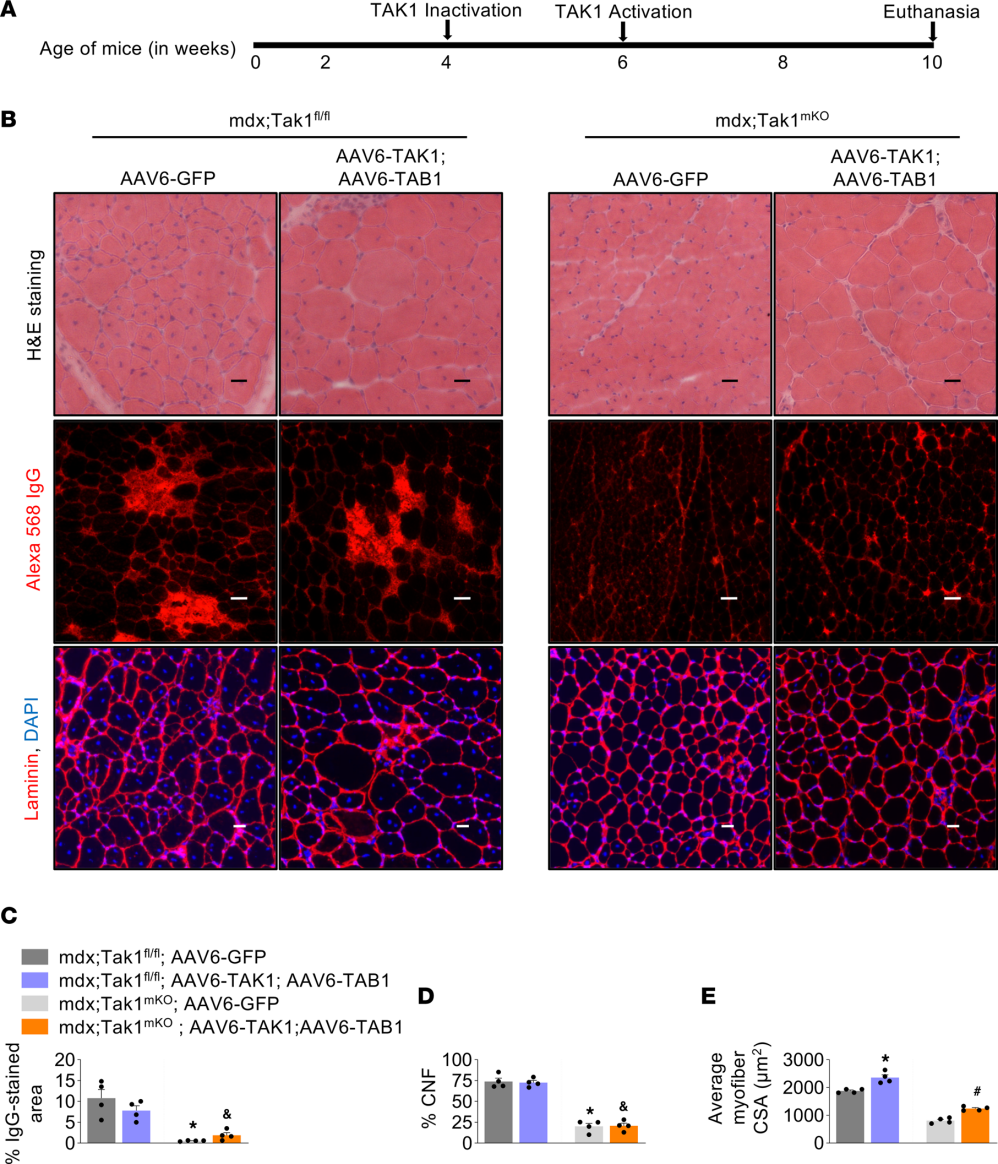
Temporal control of TAK1 expression in dystrophic muscle ameliorates histopathology. (**A**) Schematic representation showing the age of mdx mice, time of TAK1 inactivation, AAV injection, and euthanasia. (**B**) Representative photomicrographs of TA muscle sections after H&E staining, anti–mouse IgG staining, or anti–laminin staining. DAPI was used to stain nuclei. Scale bar: 50 μm. (**C**–**E**) Percentage of IgG-stained area, percentage of centronucleated fibers (CNFs), and average myofiber cross-section area (CSA) in TA muscle of mdx;Tak1^fl/fl^ and mdx;Tak1^mKO^ mice injected with AAV6-GFP or a combination of AAV6-TAK1 and AAV6-TAB1. *n* = 4 per group. Data represented as mean ± SEM and were analyzed by 2-way ANOVA followed by Tukey’s multiple-comparison test. *P ≤ 0.05, values significantly different from TA muscle of mdx;Tak1^fl/fl^ injected with AAV6-GFP. ^&^*P* ≤ 0.05, values significantly different from TA muscle of mdx;Tak1^fl/fl^ mice coinjected with AAV6-TAK1 and AAV6-TAB1. ^#^*P* ≤ 0.05, values significantly different from TA muscle of mdx;Tak1^mKO^ mice injected with AAV6-GFP.

Considering our preceding results, we speculated that TAK1 activity is temporally regulated with age in dystrophic muscle of mdx mice. TAK1 exacerbates injury during the initial myonecrotic phase. However, TAK1 also functions to maintain muscle mass and support growth. To validate our hypothesis, we devised a strategy to inactivate TAK1 at the onset of necrosis-regeneration phase and to restore TAK1 activity during the postnecrotic regenerative phase. For this experiment, we used littermate mdx;Tak1^fl/fl^ and mdx;Tak1^mKO^ mice and inactivated TAK1 at 4 weeks of age. After 2 weeks, TA muscle of mdx;Tak1^fl/fl^ and mdx;Tak1^mKO^ mice was injected with AAV6-TAK1 (1.25 × 10^10^ vg) and AAV6-TAB1 (1.25 × 10^10^ vg). Contralateral TA muscle was injected with control AAV6-GFP vector (2.5 × 10^10^ vg). After 4 weeks (i.e., at 10 weeks of age), the mice were euthanized and the TA muscle was isolated for morphometric analysis (Figure 9A). H&E staining showed that TA muscle from mdx;Tak1^mKO^ mice had substantially fewer CNFs and fewer empty areas filled with cellular infiltrates compared with TA muscle from mdx;Tak1^fl/fl^ mice (Figure 9B). Coinjection of AAV6-TAK1 and AAV6-TAB1 or with AAV6-GFP alone did not cause any noticeable change in these features in mdx;Tak1^fl/fl^ or mdx;Tak1^mKO^ mice (Figure 9B). To estimate the extent of necrotic areas, we performed immunostaining with Alexa 568–labeled mouse IgG. Results demonstrate significantly fewer numbers of IgG-filled myofibers in both GFP-expressing or TAK1- and TAB1-expressing TA muscle of mdx;TAK1^mKO^ mice compared with corresponding GFP-expressing or TAK1- and TAB1-expressing TA muscle of mdx;Tak1^fl/fl^ mice (Figure 9, B and C). Additionally, there was a significant reduction in the proportion of CNFs in both GFP-expressing or TAK1- and TAB1-expressing TA muscle of mdx;TAK1^mKO^ mice compared with corresponding GFP-expressing or TAK1- and TAB1-expressing TA muscle of mdx;Tak1^fl/fl^ mice (Figure 9, B and D). However, a significant increase in average myofiber CSA was noticeable in TA muscle coinjected with AAV6-TAK1 and AAV6-TAB1 compared with TA muscle injected with AAV6-GFP alone in mdx;Tak1^fl/fl^ mice. Moreover, the average myofiber CSA of TAK1- and TAB1-overexpressing TA muscle was significantly higher than GFP-expressing TA muscle of mdx;Tak1^mKO^ mice (Figure 9, B and E). Skeletal muscle of mdx mice contains myofibers of varying size, some with unusually large myofiber diameters compared with WT mice. Therefore, we compared the myofiber size distribution between TAK1- and TAB1-expressing TA muscle from mdx;Tak1^mKO^ mice and GFP-expressing mdx;Tak1^fl/fl^ mice with TA muscle of age-matched WT mice (Supplemental Figure 9A). Relative frequency distribution analysis showed that myofiber CSA of TA muscle from mdx;Tak1^mKO^ mice overexpressing TAK1 and TAB1 was similar to that of TA muscle of age-matched WT mice (Supplemental Figure 9B). Conversely, a proportion of myofibers in the TA muscle of GFP-expressing mdx;Tak1^fl/fl^ mice had abnormally large CSA compared with myofibers from TA muscle of age-matched WT mice (Supplemental Figure 9B). Altogether, these results suggest that tuning TAK1 expression with age alleviates dystrophinopathy in mdx mice.

## Discussion

Mutation in the dystrophin gene is present at birth, yet most DMD cases are diagnosed at 2–3 years of age when symptoms emerge (1, 50). However, the molecular and signaling mechanisms that trigger the onset and progression of myopathy in patients with DMD remain less understood. In this study, we demonstrate that TAK1 is an important regulator of dystrophic phenotype in mdx mice. TAK1 is highly activated in the skeletal muscle of young mdx mice during the initial necrotic spike, where its role is to accelerate myofiber injury and augment inflammation. Intriguingly, TAK1 also functions to maintain skeletal muscle mass and is indispensable for skeletal muscle growth in both young and adult mdx mice. Our results demonstrate that forced activation of TAK1 through overexpression of TAK1 and TAB1 promotes myofiber growth without producing any adverse effects on muscle histopathology in mdx mice.

TAK1 mediates the activation of multiple downstream signaling pathways, including NF-κB and p38 MAPK, in response to receptor-mediated events (29, 33, 51). Previous studies have shown that NF-κB is activated in skeletal muscle of mdx mice and that targeted inhibition of NF-κB in myofibers or macrophages improves dystrophic phenotype in mdx mice (21–23, 27, 28, 52). Similarly, it has been reported that the activation of p38MAPK contributes to myofiber injury in dystrophic muscle of mdx mice (24, 25). Consistent with its role in the activation of NF-κB and p38MAPK, we found that targeted inducible inactivation of TAK1 reduces muscle injury and accumulation of macrophages in dystrophic muscle of mdx mice (Figure 3, Figure 5, and Supplemental Figure 5). However, we did not find any improvement in muscle mass or function. While the exact mechanisms by which TAK1 induces muscle injury during peak necrotic phase remain unknown, it is possible that TAK1-mediated signaling in myofibers influences the function of various interstitial cells through paracrine mechanisms to promote myofiber necrosis. Moreover, it is also plausible that reduced muscle injury observed in *Tak1*-deficient mdx mice during peak necrotic phase is a result of reduced myofiber growth or size. Indeed, our experiments demonstrate that myofiber size is significantly reduced upon inactivation of TAK1 in both young and adult mdx mice (Figures 2 and 7). Interestingly, our results also demonstrate that forced activation of TAK1 through AAV-mediated overexpression of TAK1 and TAB1 does not produce any adverse effect on muscle histopathology but promotes growth of myofibers in adult mdx mice (Figures 8 and 9). These results are consistent with our findings in WT mice, where we demonstrated that targeted inactivation of TAK1 causes muscle atrophy (35, 36), whereas its forced activation promotes muscle growth potentially through stimulating protein synthesis and improving the stability of NMJs (37).

A recent report showed that TAK1 is activated in skeletal muscle of patients with DMD and that inhibition of TAK1 ameliorates dystrophic phenotype in mdx mice (38). In contrast, we found that muscle-specific inactivation of TAK1 reduces grip strength and muscle contractile function in mdx mice (Figures 2 and 7). Although the exact reasons for this discrepancy in muscle phenotype in the 2 studies remain unknown, it might be attributed to the different approaches that were used to inhibit TAK1 activity. While the published report shows a small reduction in TAK1 levels after injection of AAV-TAK1 shRNA (38), our genetic approach ensures a drastic reduction in the levels of TAK1 protein in dystrophic muscle of mdx mice (Figures 5 and 7). Additionally, it was reported that pharmacological inhibition or knockdown of TAK1 using shRNA ameliorates fibrosis in mdx mice (38). However, we found that myofiber-specific ablation of TAK1 does not affect fibrosis in mdx mice (Supplemental Figure 7, D and E). Moreover, unlike in patients with DMD where necrotic myofibers are replaced by noncontractile fibrotic tissue, regenerative myogenesis is activated in mdx mice and fibrosis is a secondary effect observed in the diaphragm of older mdx mice (53). We speculate that, in addition to myofibers, a pharmacological or shRNA-mediated knockdown approach can delete TAK1 in multiple cell types, leading to the discrepant phenotype. Indeed, a recent report shows that TAK1 controls the fibrogenic differentiation of fibro/adipogenic progenitor (FAP) cells in skeletal muscle and that pharmacological inhibition of TAK1 by 5-Z-7 Oxozeaenol considerably reduces gene expression of fibrotic molecules, such as Collagen 1a1 (Col1a1), connective tissue growth factor (CTGF), periostin (Postn), smooth muscle actin (Acta2), and fibronectin 1 (Fn1) in FAPs (54). While we have used genetic mouse models, both groups of mice were fed tamoxifen-containing chow starting from the last day of tamoxifen injection, for the entire duration of the experiment. It remains unknown whether tamoxifen differentially affects muscle phenotype in mdx;Tak1^fl/fl^ and mdx;Tak1^mKO^ mice.

Even though overall muscle mass and function were diminished, we observed that muscle-specific inactivation of TAK1 in young mdx mice inhibits myofiber injury (Figures 2 and 3). Temporal increase in TAK1 levels in GA muscle of mdx mice during the necrotic phase (~4 weeks) is accompanied with a pronounced increase in RIPK1 phosphorylation at S321 residue (Figure 1E), which is known to induce necroptosis and tissue damage (40). While destabilization of sarcolemma is the major mechanism for myofiber injury, a recent report has shown that the necroptosis pathway is also activated in the skeletal muscle of mdx mice and inhibition of necroptosis alleviates muscle damage (41). Our experiments demonstrate that inactivation of TAK1 substantially inhibits phosphorylation of RIPK1 in dystrophic muscle of 5- and 8-week-old mdx mice (Figure 5C). Since RIPK1 is involved in cell demise through necroptosis, we speculate that TAK1 might be inducing myonecrosis during the peak necrotic phase through necroptosis as well. Interestingly, forced activation of TAK1 through overexpression of TAK1 and TAB1 does not cause myofiber injury in skeletal muscle of WT mice (37) or exacerbate injury in dystrophic muscle of mdx mice (Figures 8 and 9). While physiological significance of TAK1-mediated phosphorylation of RIPK1 remains unknown, TAK1 and RIPK1 also mediate the activation of multiple signal transduction pathways involved in inflammatory response. Indeed, inflammatory immune cells contribute significantly to muscle cell death during the peak necrotic phase in mdx mice (5). Therefore, it is possible that inflammatory molecules present during the peak necrotic phase stimulate TAK1 activity, which further exacerbates inflammation, leading to myofiber injury in mdx mice. Inactivation of TAK1 during the peak necrotic phase may reduce muscle damage through the inhibition of inflammatory immune response and/or necroptosis.

We have previously reported that inducible inactivation of TAK1 leads to the activation of the UPS and autophagy in skeletal muscle of WT mice (37). Our results in the present study demonstrate that the components of UPS and autophagy are also increased in skeletal muscle of mdx mice following TAK1 inactivation (Figure 6), suggesting that TAK1 regulates common proteolytic systems in normal and dystrophic muscle. Autophagy is an important physiological mechanism through which damaged organelles are cleared in mammalian cells (55). Targeted inhibition of autophagy causes severe myopathy in WT mice (56, 57). Interestingly, autophagy is repressed in skeletal muscle of mdx mice and in patients with DMD (58). Indeed, recent reports have shown that restoring autophagy using pharmacological activators improves dystrophic phenotype in preclinical animal models of DMD, including mdx mice (12, 59). Our results suggest that activation of autophagy following TAK1 inactivation could be another mechanism for reduced muscle injury and improvement in muscle structure in young mdx mice (Figure 6).

An important observation of the present study is that forced activation of TAK1 improves muscle growth in mdx mice after the initial wave of myofiber necrosis. This is evidenced by our finding that overexpression of TAK1 and TAB1 stimulates myofiber hypertrophy in 12-week-old mdx mice. Moreover, increased activation of TAK1 also improves myofiber size and normalizes fiber size distribution in young mdx mice. Similar to skeletal muscle of WT mice (37), our experiments suggest that stimulation of TAK1 promotes muscle growth through augmentation of protein translation in mdx mice.

In summary, our present study provides initial evidence that activation of TAK1 supports myofiber growth in mdx mice. One of the limitations of our study is that we have employed mdx mice where necrotic phase is well delimited. While mdx mice demonstrate many changes similar to patients with DMD, the mice do not entirely recapitulate the disease progression and fibrosis. Certainly, more investigations are needed in other mouse models, including DBA/2J-mdx mice, and higher vertebrates, such as the golden retriever muscular dystrophy model, to test whether overexpression of TAK1 and TAB1 ameliorates dystrophinopathy and improve muscle mass. Similar to TAK1, inhibition of myostatin promotes muscle growth in animal models. However, myostatin inhibition therapy failed to show modification of disease course in patients with DMD in clinical trials (60). Therefore, it will be important to investigate whether prolonged supraphysiological activation of TAK1 augments growth of dystrophin-deficient myofibers in dystrophic muscle independently of signaling from TGF-β family members. Nonetheless, our study demonstrates that TAK1 promotes myofiber growth in mdx mice. Proper regulation of TAK1 can be a potential therapeutic approach to ameliorate disease progression and to improve the life spans of patients with DMD.

## Methods

### Animals.

Skeletal muscle–specific Tak1^mKO^ were generated by crossing HSA-MCM mice (The Jackson Laboratory; Tg[ACTA1-cre/Esr1*]2Kesr/J) with Tak1^fl/fl^ mice as described (29, 35). Tak1^mKO^ mice were crossed with mdx mice to generate littermate mdx;Tak1^fl/fl^ and mdx;Tak1^mKO^ mice. All the mice used in this study, including mdx mice, were on C57BL/6 background. Genotypes of mice were determined by performing PCR from tail DNA using AccuStart II PCR genotyping kit (Quantabio). The mice were housed in a 12-hour light-dark cycle and given water and food ad libitum. Inactivation of TAK1 kinase through Cre-mediated recombination in mdx;Tak1^mKO^ mice was accomplished by daily i.p. injections of tamoxifen (Sigma-Aldrich, 75 mg/kg body weight) for 4 consecutive days. Thereafter, the mice were fed a tamoxifen-containing chow (250 mg/kg; Harlan Laboratories) for the duration of the experiment. Littermate mdx;Tak1^fl/fl^ mice (controls) were also treated with tamoxifen and fed tamoxifen-containing chow. For intramuscular delivery of AAV, mice were anesthetized with isoflurane (Covetrus) and 2.5 × 10^10^ AAV vector genomes (in 30 μL PBS) were injected in the TA muscle of mice, as described in ref. 37.

### AAV vectors.

AAVs (serotype 6) were custom generated by Vector Biolabs. The AAVs expressed either GFP, Tak1 (no. BC006665), or Tab1 (no. BC054369) genes under a ubiquitous CMV promoter.

### Skeletal muscle injury.

Left TA muscle of Tak1^fl/fl^ and Tak1^mKO^ mice was injected with 100 μL of 10 μM cardiotoxin (CalBiochem) dissolved in PBS. Five days after injury, the mice were euthanized and TA muscle was isolated for morphometric analysis.

### Grip strength measurements.

To measure total 4-limb grip strength of mice, a digital grip-strength meter (Columbus Instruments) was used. Before performing tests, mice were acclimatized for 5 minutes. The mouse was allowed to grab the metal pull bar with all 4 paws. The tail of the mouse was then gently pulled backward in the horizontal plane until the mouse could no longer grasp the bar. The force produced at the time of release was recorded as the peak tension. Each mouse was tested 5 times with a 20- to 40-second break between tests. The average peak tension from the 5 best attempts and maximum peak tension normalized against total body weight was defined as average grip strength.

### In vivo muscle functional assay.

In vivo force measurements of the posterior lower leg muscles were conducted using the 1300A 3-in-1 Whole Animal System (Aurora Scientific) to evoke plantarflexion as described (42). In brief, the mice were anesthetized with isoflurane and placed on the isothermal stage. Optimal muscle length (Lo) that allows maximal isometric twitch force (Pt) was determined by a sequence of twitch contractions at 150 Hz every 1 second, with small varying changes to muscle tension and current. After muscle optimization, an average specific twitch force (sPt) was generated from 5 stimulations at 150 Hz. To obtain maximum isometric tetanic force (Po), muscles were stimulated from 25–300 Hz in 25 Hz intervals, resulting in 12 specific tetanic forces (sPo). There was a 30-second delay between the first 2 recordings and a 1-minute delay between subsequent recordings to allow sufficient muscle recovery. Following the 300 Hz stimulation, a baseline recording was obtained. Then, the muscles were fatigued at 150 Hz with 1 contraction per second for 180 seconds. Force recordings for obtained specific isometric sPt (mN/m) and sPo (mN/m) were normalized by total mouse body weight. For all experiments, a prestimulus and poststimulus baseline of 200 ms was recorded to establish a baseline recording. For all experiments, a 0.2 ms pulse width was used. Current was adjusted on an individual basis to evoke the maximum amount of force. Contractile events were recorded using the ASI 611A Dynamic Muscle Analysis software (Aurora Scientific) at a sampling rate of 2,000 Hz. Force (mN/m) and corresponding integral values for sPt (mN/m/s) and sPo (mN/m/s) were calculated using the accompanying.

### CK assay.

Levels of CK in serum were determined using a commercially available kit (Stanbio CK Liqui-UV Test, Stanbio Laboratory) following a protocol suggested by the manufacturer.

### Histology and morphometric analysis.

We performed H&E staining (Sigma-Aldrich) or Sirius red staining (StatLab) protocol on transverse diaphragm, TA, and soleus muscle sections to visualize muscle structure or fibrosis, respectively. In brief, individual hind limb muscle was isolated, flash frozen in liquid nitrogen, and sectioned using a microtome cryostat (Cryostar NX-50, Thermo Fisher Scientific). For the assessment of gross morphology, 8–10 μm thick transverse muscle sections were stained with H&E dye. Stained sections were visualized and captured using an inverted microscope (Nikon Eclipse Ti-2e microscope) and a digital camera (Digital Sight DS-FI3, Nikon) at room temperature. Finally, images of the muscle sections were used for quantification of number of CNFs, total number of myofibers, and average CSA. Necrotic area in H&E-stained sections was determined by measuring percentage area filled with cellular infiltrate in the whole muscle section. The extent of fibrosis in muscle transverse sections was determined using a Picrosirius red staining kit following a protocol suggested by manufacturer (StatLab). Morphometric analyses were quantified using Fiji software (NIH).

### IHC.

For IHC studies, muscle sections were blocked in 1% BSA (Sigma-Aldrich) in PBS for 1 hour and incubated with primary antibodies anti-Pax7 (1:10, Developmental Studies Hybridoma Bank [DSHB], University of Iowa, Iowa City, Iowa, USA), anti–E-MyHC (1:200, DSHB, University of Iowa), and anti-laminin (1:100, MilliporeSigma) in blocking solution at 4°C overnight under humidified conditions. For F4/80 staining, the sections were incubated with anti-F4/80 monoclonal antibody (BM8) and Per CP–cyanine 5.5 in blocking buffer for 2 hours at room temperature. The sections were washed briefly with PBS before incubation with secondary Alexa Fluor tagged secondary antibody for 1 hour at room temperature and then washed 3 times for 5 minutes with PBS. Next, the sections were stained with DAPI (Thermo Fisher Scientific), and the slides were mounted using a nonfluorescing aqueous mounting medium (Vector Laboratories). For fiber typing, transverse sections of soleus or TA muscle were blocked with 2% BSA for 45 minutes. The slides were then incubated with monoclonal antibodies against type I, IIa, and IIb MyHC isoforms using clone BA-D5, SC-7, and BF-F3, respectively (DSHB) for 2 hours at room temperature. After PBS wash, the sections were incubated with goat anti–mouse IgG2b conjugated with Alexa Fluor 350, goat anti–mouse IgG1 conjugate with Alexa-568, and goat anti–mouse IgM conjugated with Alexa Fluor 488 secondary antibodies for 45 minutes (Supplemental Table 1). Finally, the slides were mounted using a nonfluorescing aqueous mounting medium (Vector Laboratories). Damaged/permeabilized fibers in muscle cryosections were identified by immunostaining with Alexa Fluor 488– or Alexa Fluor 568–conjugated anti–mouse IgG. Images were captured using Nikon Eclipse Ti-2e microscope (Nikon) attached with a Prime BSI Express Scientific CMOS (sCMOS) camera and Nikon NIS Elements AR software. Pseudo colors were used for representation of images. Images were stored as TIFF files, and contrast levels were equally adjusted using Adobe Photoshop CS6 software. The antibodies used are provided in Supplemental Table 1.

### Western blot.

Relative levels of various proteins in skeletal muscle tissue of mdx mice were estimated by Western blot as previously described (32). Briefly, skeletal muscle tissues were washed with PBS and homogenized in ice-cold lysis buffer consisting of the following: 50 mM Tris-Cl (pH 8.0), 200 mM NaCl, 50 mM NaF, 1 mM DTT, 1 mM sodium orthovanadate, 0.3% IGEPAL, and protease inhibitors (Halt Protease Inhibitor Cocktail, Thermo Fisher Scientific). Approximately 100 μg protein was resolved in each lane on 10%–12% SDS-polyacrylamide gels, electrotransferred onto nitrocellulose membranes, and probed using primary antibody. Signal detection was performed by an enhanced chemiluminescence detection reagent (Bio-Rad). Approximate molecular masses were determined by comparison with the migration of prestained protein standards (Bio-Rad). Quantitative estimation of the bands’ intensity was performed using ImageJ software (NIH). The antibodies used are provided in Supplemental Table 1. The images of uncropped gels are presented in Supplemental Figure 10.

### RNA isolation and qPCR assay.

Total RNA isolation from muscle tissues and qPCR was performed as previously described (61, 62). Primers for qPCR were designed using Vector NTI software, and their sequence has been presented in Supplemental Table 2.

### Statistics.

We calculated sample size using a size power analysis method for a priori determination based on the SD, and the effect size was previously obtained using the experimental procedures employed in the study. For animal studies, we estimated sample size from expected number of mdx;Tak1^mKO^ mice and mdx;Tak1^fl/fl^ mice. We calculated the minimal sample size for each group as 8 animals. For some experiments, 3–4 animals were found sufficient to obtain statistical differences. Littermate animals with same sex and same age were employed to minimize physiological variability and to reduce SEM. The exclusion criteria for animals were established in consultation with IACUC members and experimental outcomes. In case of death, skin injury, sickness, or weight loss of > 10%, the mouse was excluded from analysis. Muscle tissue samples were not used for analysis in cases such as freeze artifacts on histological section or in failure of extraction of RNA or protein of suitable quality and quantity. Animals from different breeding cages were included by random allocation to the different experimental groups. Animal experiments were blinded using number codes until the final data analyses were performed. Results are expressed as mean ± SEM. Statistical analyses used 2-tailed Student’s *t* test or 1- or 2-way ANOVA followed by Tukey’s multiple-comparison test. *P* < 0.05 was considered statistically significant.

### Study approval.

All animal procedures were conducted in strict accordance with the institutional guidelines and were approved by the IACUC and Institutional Biosafety Committee of the University of Houston (IACUC no. 201900043).

### Data and materials availability.

All data are available in the main text or the supplemental materials.

## Author contributions

Conceptualization was contributed by AR, MW, and AK. Methodology was contributed by AR, TEK, ASJ, MTDS, and KM. Investigation was contributed by AR, TEK, ASJ, MTDS, and KM. Visualization was contributed by AR, TEK, and MTDS. Funding acquisition was contributed by AK. Project administration was contributed by AR. Supervision was contributed by AK. Writing of the original draft was contributed by AR. Review and editing of the manuscript were contributed by AR, ASJ, MTDS, MW, and AK.

## Supplementary Material

Supplemental data

## Version 1 04/18/2023

In-Press Preview

## Version 2 05/22/2023

Electronic publication
