# Supplementary material for: Targeted regulation of TAK1 counteracts dystrophinopathy in a DMD mouse model
Source: JCI Insight. 2023 May 22;8(10):e164768. doi: 10.1172/jci.insight.164768 (PMC10322678; doi:10.1172/jci.insight.164768)
Supplement: Supplemental data [file jciinsight-8-164768-s141.pdf]

Supplemental Data File

Targeted regulation of TAK1 counteracts dystrophinopathy in a DMD mouse model

By

Anirban Roy, Tatiana E. Koike, Aniket S. Joshi, Meiricris Tomaz da Silva, Kavya Mathukumalli, Mingfu Wu, and Ashok Kumar

This file contains Figures S1-S10 and Tables S1 and S2

Supplemental Figure S1

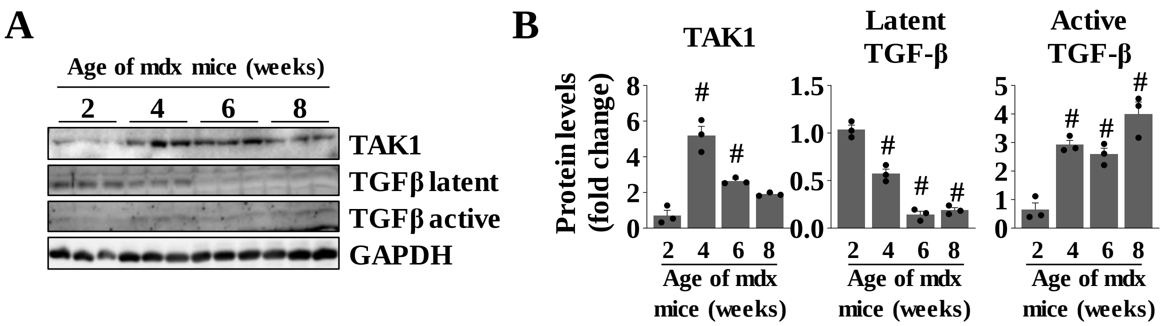

Fig. S1. Levels of TAK1 and latent and active TGF-β protein in dystrophic muscle of mdx mice. (**A**) Western blot and (**B**) densitometry analysis showing levels of TAK1 and latent and active TGF-β protein in TA muscle of mdx mice at indicated age. n=3. #p≤0.05, values significantly different from TA muscle of 2-week-old mdx mice by one-way ANOVA followed by Tukey's multiple comparison test.

Supplemental Figure S2

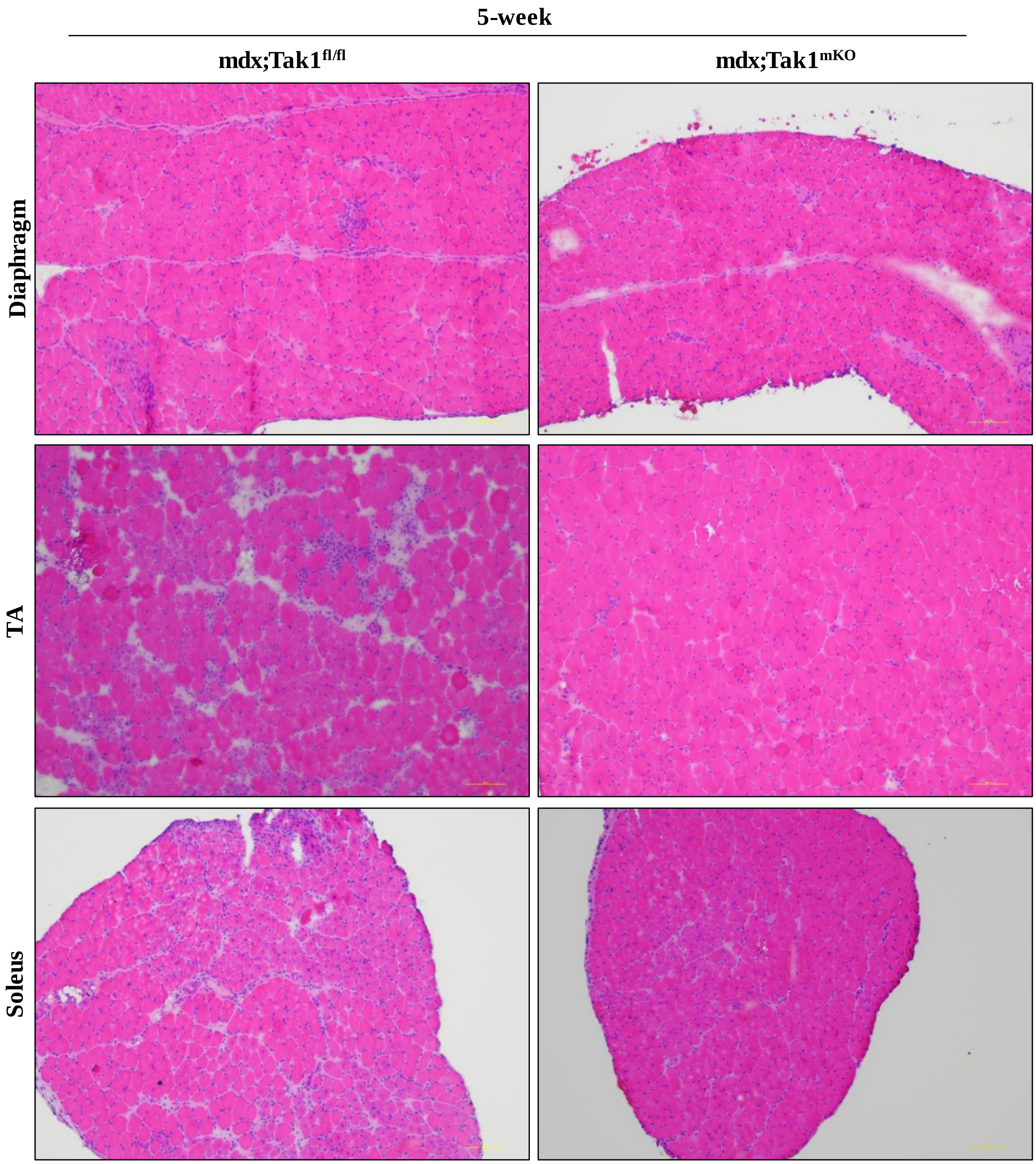

Fig. S2. TAK1 inactivation inhibits myonecrosis in young mdx mice. Uncropped 10X images of transverse sections of diaphragm, TA, and soleus muscle in 5-week old mdx;Tak1$^{fl/fl}$ and mdx;Tak1^{mKO} mice. Scale bar, 100 µm.

Supplemental Figure S3

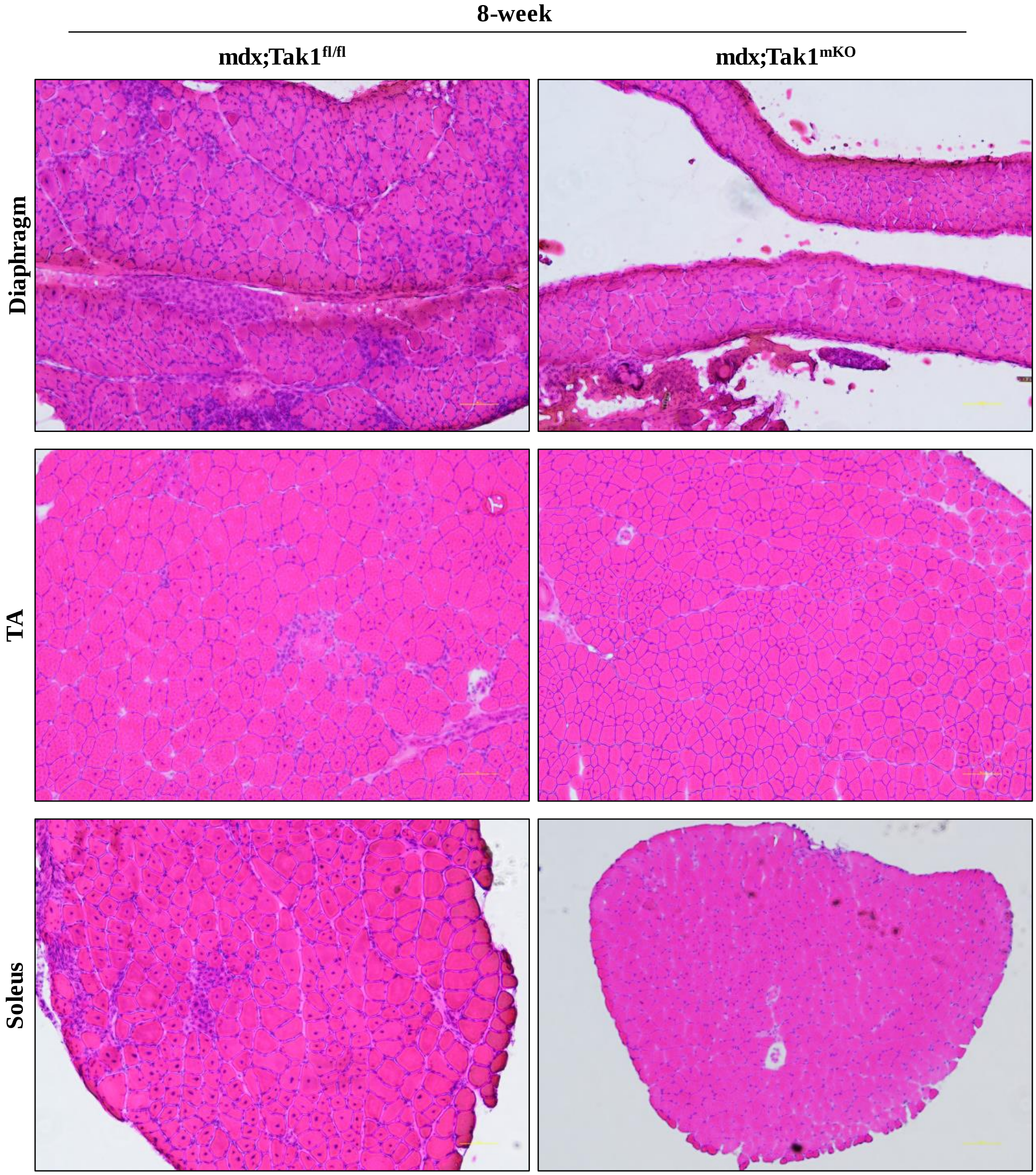

Fig. S3. TAK1 inactivation inhibits myonecrosis and myofiber size in mdx mice. Uncropped 10X images of transverse sections of diaphragm, TA and soleus muscle in 8-week old mdx;Tak1$^{fl/fl}$ and mdx;Tak1^{mKO} mice. Scale bar, 100 µm.

Supplemental Figure S4

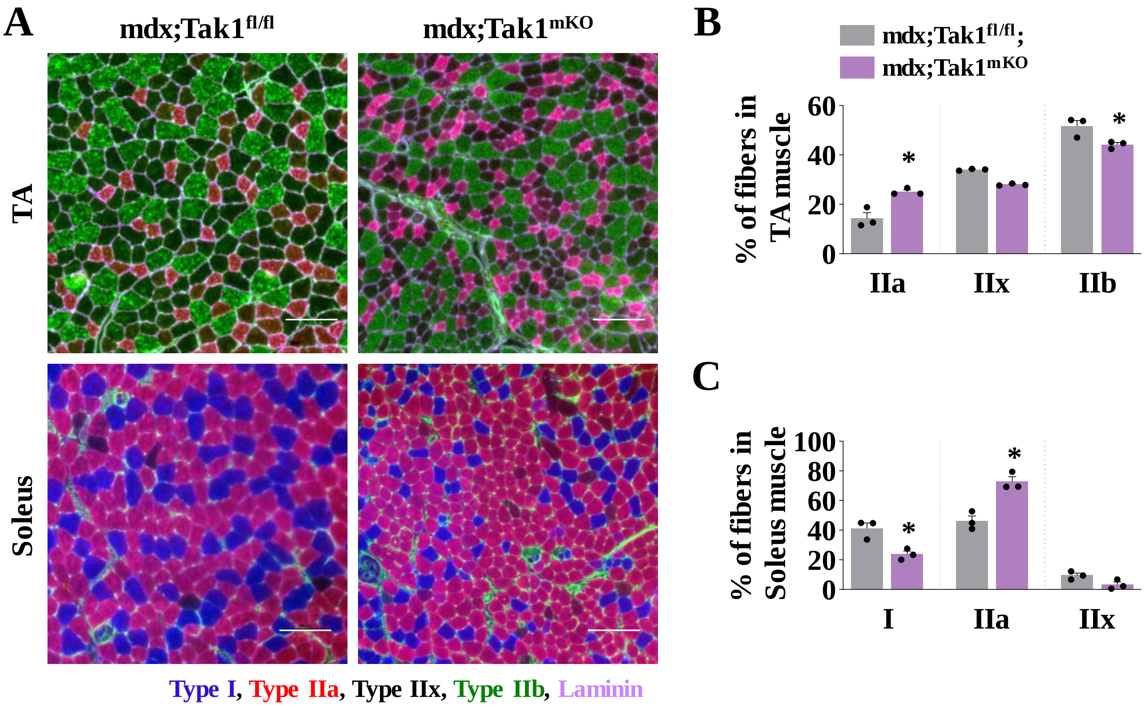

Fig. S4. Inactivation of TAK1 increases proportion of oxidative myofibers in mdx mice. 4-week-old mdx;Tak1$^{fl/fl}$ and mdx;Tak1^{mKO} mice were treated with tamoxifen and analyzed at the age of 8 weeks. (**A**) Representative photomicrographs of transverse sections from TA and soleus muscle after triple immunostaining for MyHC I, -IIa, and -IIb protein. Scale bars: 100 µm. Quantitative analysis showing percentage of each fiber type in (**B**) TA and (**C**) soleus muscle. n=3. Data represented as mean ± SEM. *p≤0.05, values significantly different from mdx;Tak1$^{fl/fl}$ mice by unpaired Student *t* test.

Supplemental Figure S5

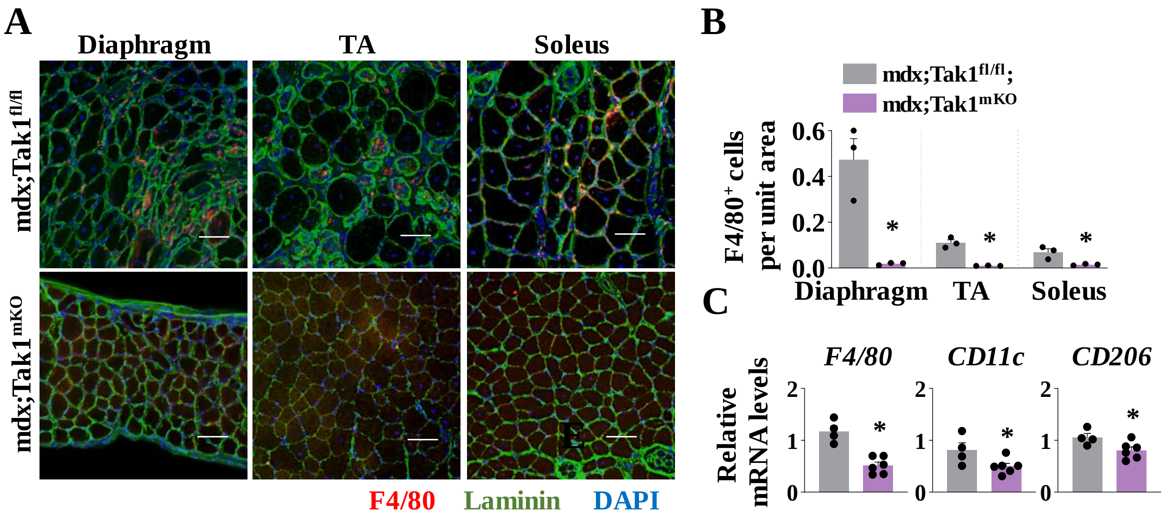

Fig. S5. Inactivation of TAK1 suppress macrophage infiltration in dystrophic muscle of mdx mice. (**A**) Representative photomicrographs of transverse sections of diaphragm, TA, and soleus muscle of 8-week-old mdx;Tak1$^{fl/fl}$ and mdx;Tak1^{mKO} mice immunostained for F4/80 antigen and laminin protein. DAPI was used to visualize nuclei. **(B)** Quantification of F4/80^{+} cells per unit area in diaphragm, TA, and soleus muscle of 8-week-old mdx;Tak1$^{fl/fl}$ and mdx;Tak1^{mKO} mice. n=3. (**C**) Relative mRNA levels of F4/80, CD11c, and CD206 in GA muscle of 8-week old mdx;Tak1$^{fl/fl}$ and mdx;Tak1^{mKO} mice. n=4-6 mice per group. Data represented as mean ± SEM. *p≤0.05, values significantly different from mdx;Tak1$^{fl/fl}$ mice by unpaired Student *t* test.

Supplemental Figure S6

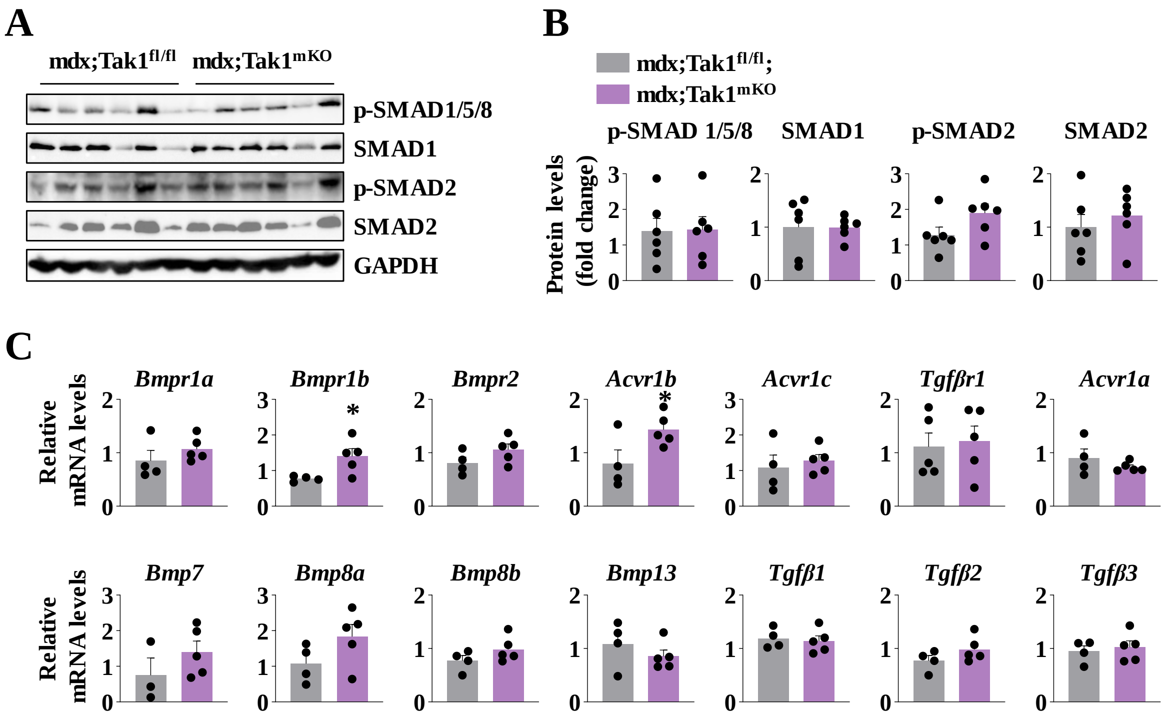

Fig. S6. Effects of inactivation of TAK1 on Smad signaling in mdx mice. 4-week-old mdx;Tak1$^{fl/fl}$ and mdx;Tak1^{mKO} mice were treated with tamoxifen and analyzed at the age of 8 weeks. (**A**) Western blot and (**B**) densitometry analysis showing levels of p-Smad 1/5/8, Smad1, p-Smad2, Smad2, and unrelated protein GAPDH in GA muscle of 8-week-old mdx;Tak1$^{fl/fl}$ and mdx;Tak1^{mKO} mice. n=6 in each group. (**C**) qRT-PCR showing relative mRNA levels of *Bmpr1a, Bmpr1b, Bmpr2, Acvr1b, Acvr1c, Tgfbr1, Acvr1a, Bmp7, Bmp8a, Bmp8b, Bmp13, tgfb1, Tgfb2, and Tgfb3* in GA muscle of 8-week-old mdx;Tak1$^{fl/fl}$ and mdx;Tak1^{mKO} mice. n=3-5 per group. Data represented as mean ± SEM. *p≤0.05, values significantly different from mdx;Tak1$^{fl/fl}$ mice by unpaired Student *t* test.

Supplemental Figure S7

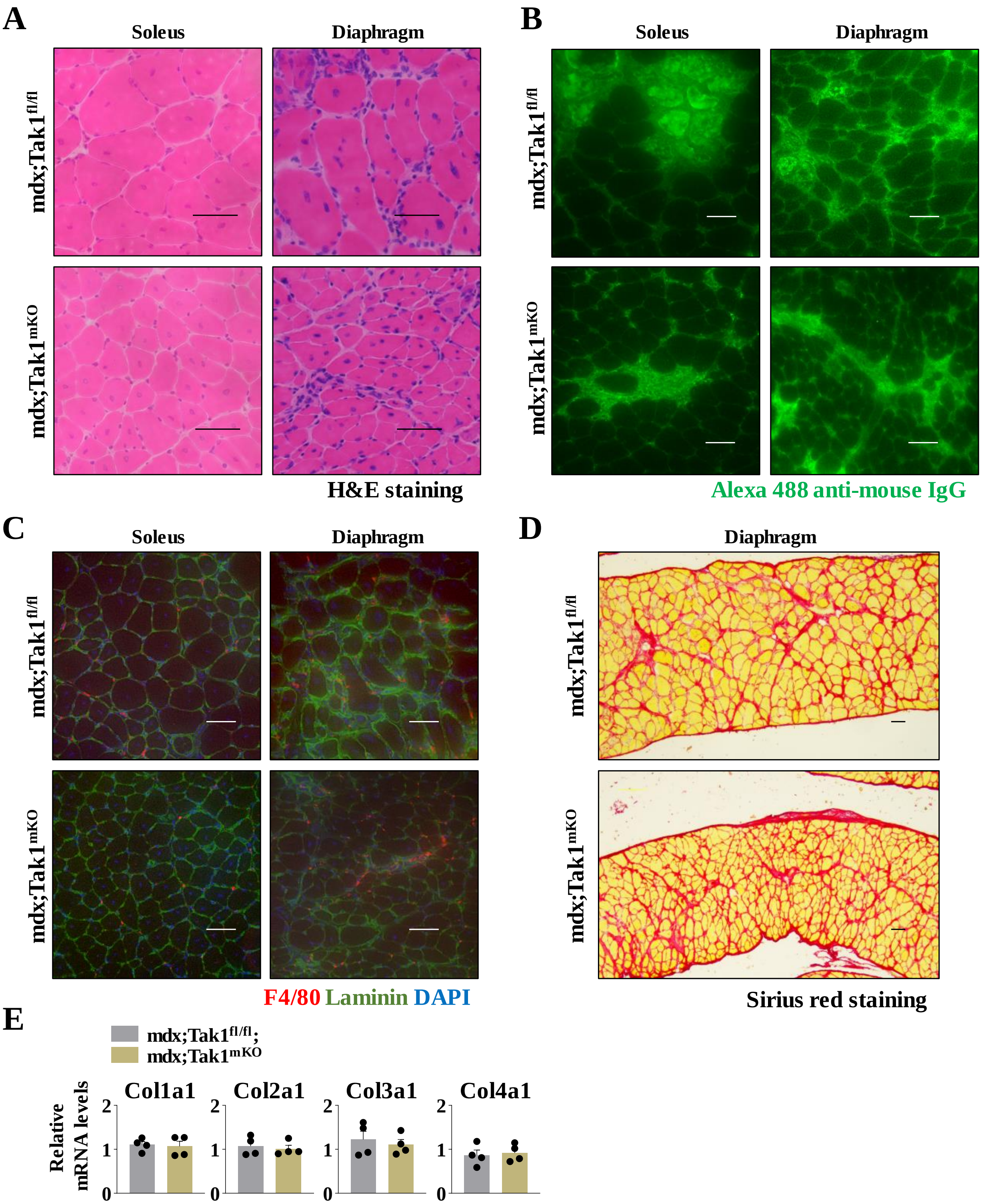

Fig. S7. TAK1 inactivation causes muscle wasting in adult mdx mice. 11.5-week-old littermate mdx;Tak1$^{fl/fl}$ and mdx;Tak1^{mKO} mice were treated with tamoxifen and analyzed at the

age of 16 weeks. Transverse sections of soleus muscle and diaphragm of mdx;Tak1$^{fl/fl}$ and mdx;Tak1^{mKO} mice after performing (**A**) H&E staining, (**B**) immunostaining with Alexa488-labelled anti-mouse IgG, and (**C**) co-immunostaining with anti-F4/80 and anti-laminin. Nuclei were stained with DAPI. (**D**) Sirius red staining of diaphragm sections of mdx;Tak1$^{fl/fl}$ and mdx;Tak1^{mKO} mice. Scale bar, 50 µm.. (**E**) Relative mRNA levels of *Col1a1*, *Col2a1*, *Col3a1*, and *Col4a1* in GA muscle of 16-week-old mdx;Tak1$^{fl/fl}$ and mdx;Tak1^{mKO} mice. n=4. Data represented as mean ± SEM. *$p \leq 0.05$, values significantly different from GA muscle of 16-week-old mdx;Tak1$^{fl/fl}$ mice by Student's t test.

Supplemental Figure S8

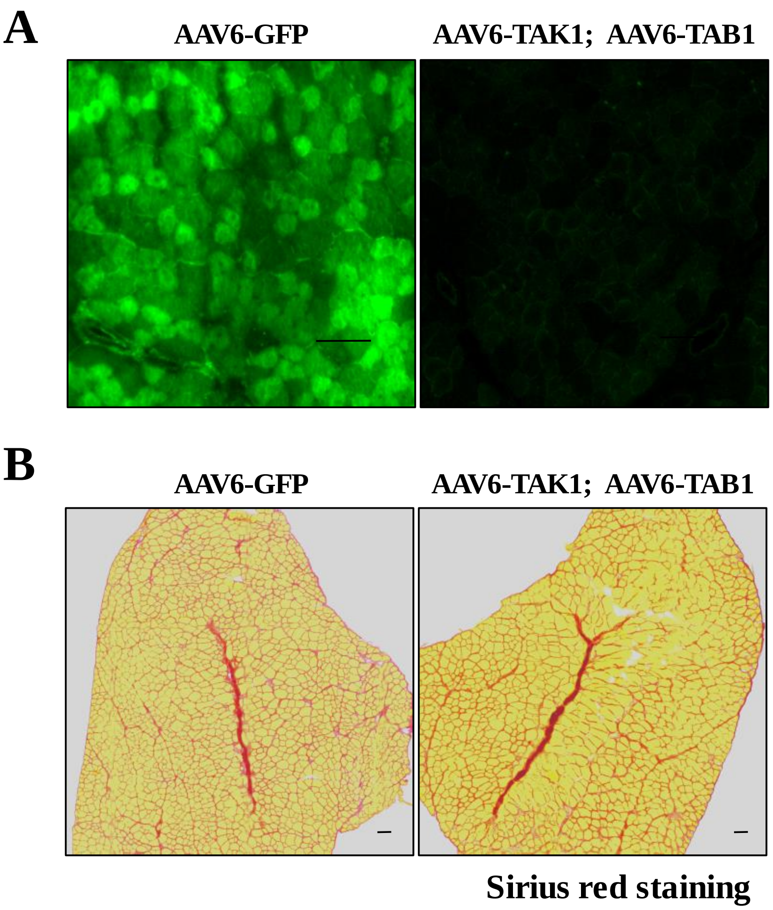

Fig. S8. Overexpression of TAK1 and TAB1 does not affect collagen deposition in dystrophic muscle of mdx mice. Left side TA muscle of 12-week-old mdx mice was given intramuscular injection of AAV6-TAB1 (1.25×10^{10} vg) and AAV6-TAK1 (1.25×10^{10} vg) while the contralateral right TA muscle was injected with AAV6-GFP (2.5×10^{10} vg) particles. After 28 days, the mice were euthanized and the TA muscle was isolated followed by generating transverse sections. (**A**) Expression of GFP in TA muscle of mdx mice injected with AAV6-GFP. Scale bar, 100 µm. (**B**) Sirius red staining showing collagen deposition in TA muscle of mdx mice injected with AAV6-GFP or a combination of AAV6-TAK1 and AAV6-TAB1. Scale bar, 100 µm.

Supplemental Figure S9

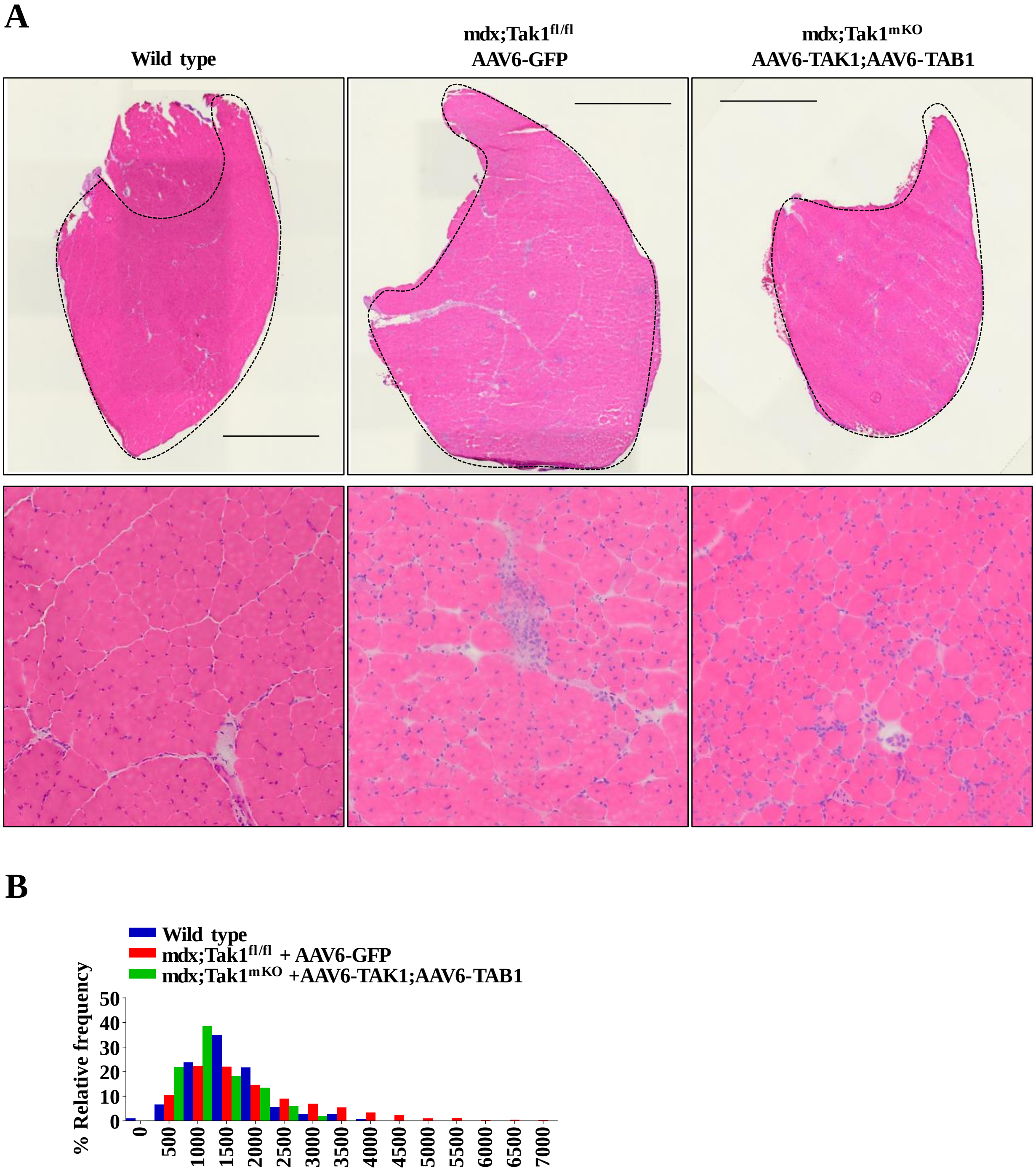

Fig. S9. Temporal regulation of TAK1 improves myopathy in mdx mice. 4-week-old mdx;Tak1$^{fl/fl}$ and mdx;Tak1^{mKO} mice were treated with tamoxifen for four days. At 6 weeks, the left side TA muscle was given intramuscular injection of AAV6-TAB1 (1.25×10^{10} vg) and AAV6-TAK1 (1.25×10^{10} vg) while the contralateral right TA muscle was injected with AAV6-

GFP (2.5×10^{10} vg) particles. At the age of 10 weeks, the mice were euthanized and the TA muscle was harvested and analyzed. (**A**) H&E-stained transverse sections of whole TA muscle (top panel) and magnified inset (bottom panel) of age matched wt mice, mdx;Tak1$^{fl/fl}$ mice injected with AAV6-GFP, and mdx;Tak1^{mKO} mice co-injected with AAV6-TAK1 and AAV6-TAB1. Scale bar, 1000 µm. (**B**) Relative frequency distribution showing myofiber CSA of wild type, mdx;Tak1$^{fl/fl}$ mice injected with AAV6-GFP, and mdx;Tak1^{mKO} mice co-injected with AAV6-TAK1 and AAV6-TAB1.

Supplemental Figure S10

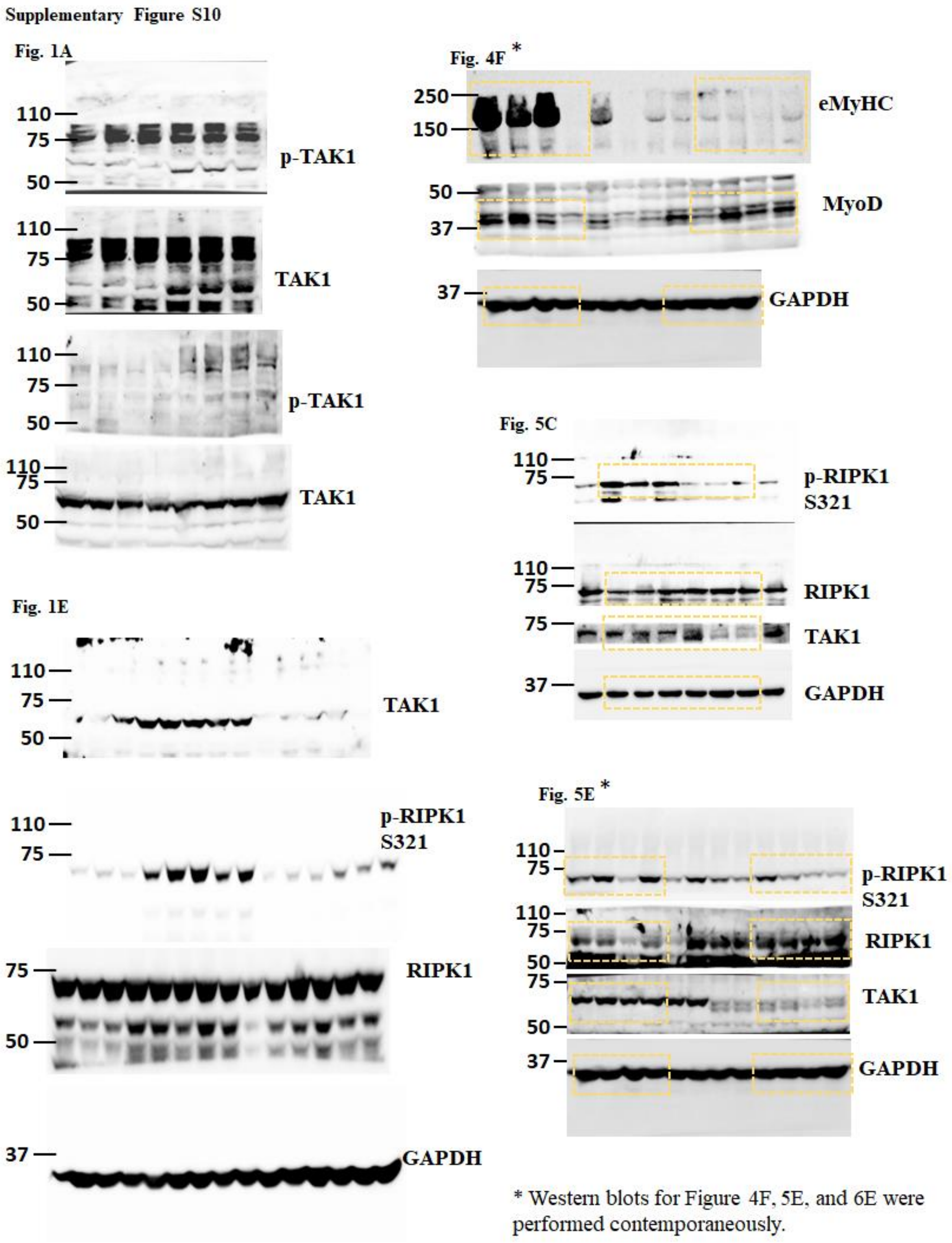

Fig. S10. Uncropped images of immunoblots. Raw immunoblots and bands shown in original figures are marked here.

Supplemental Figure S10 contd.

Fig. 6B

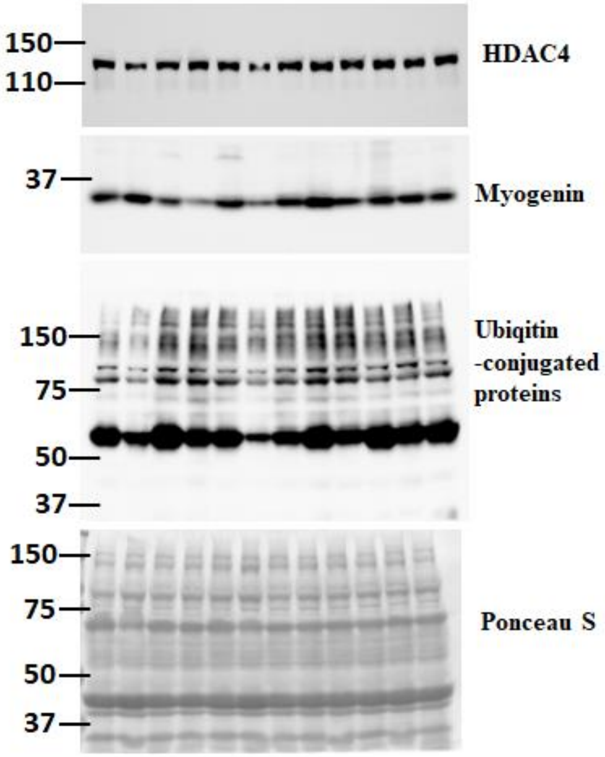

Fig. 6E *

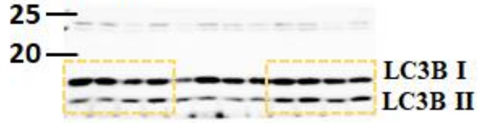

Fig. 7H

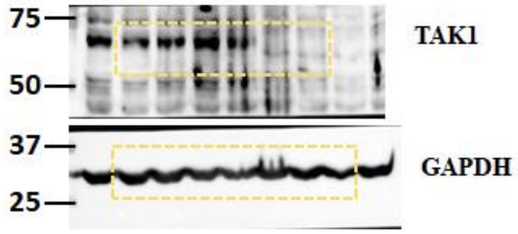

Fig. 8G

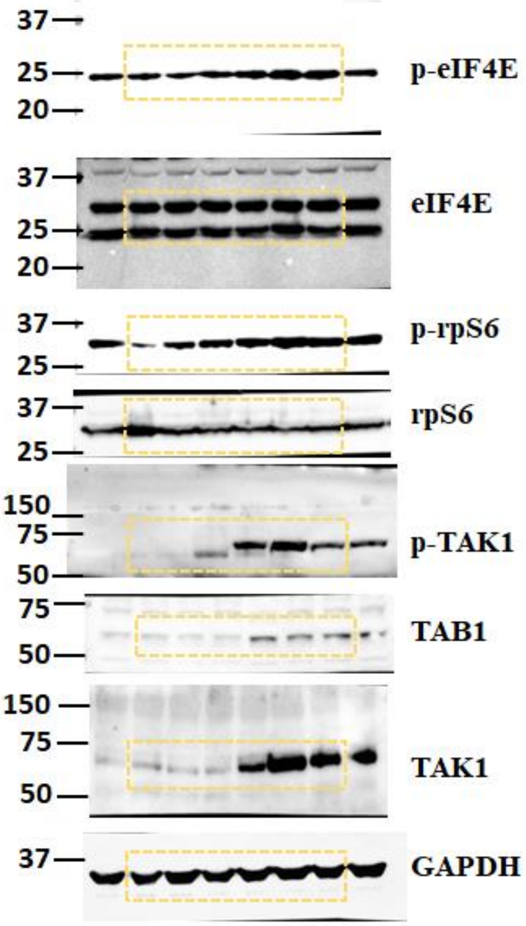

* Western blots for Figure 4F, 5E, and 6E were performed contemporaneously.

Supplemental Figure S10 contd.

Supplemental Fig. S1A

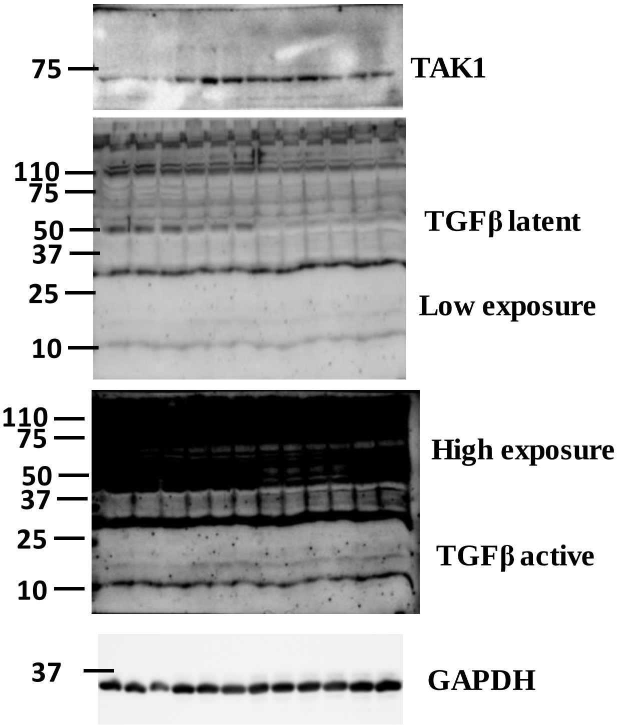

Supplemental Fig. S6A

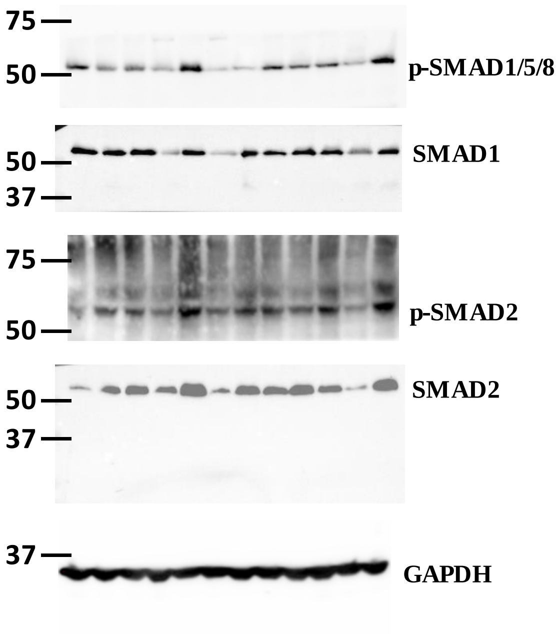

Table S1. Antibodies used in the study.

**Antibody**	**Source and Catalog no.**
Monoclonal rabbit-anti-total-TAK1	Cell Signaling Technology # 5206
Monoclonal rabbit-anti-phospho-TAK1	Invitrogen #MA5-15073
Monoclonal mouse-anti-eMyHC	DSHB Cat# F1.652
Polyclonal rabbit-anti-MyoD	Santa Cruz Biotechnology sc-304
Monoclonal rabbit-anti-GAPDH	Cell Signaling Technology # 2118
Monoclonal rabbit-anti-phospho-RIPK1	Cell Signaling Technology, # 83613
Monoclonal rabbit-anti-RIPK1	Cell Signaling Technology # 3493
Monoclonal rabbit-anti-HDAC4	Cell Signaling Technology # 7628
Monoclonal mouse-anti-Ubiquitin	Santa Cruz Biotechnology, sc-8017
Monoclonal rabbit-anti-LC3B	Cell Signaling Technology, # 3868
Monoclonal mouse-anti-myogenin	DSHB Cat#F5D
Polyclonal rabbit-anti-phospho-eIF4E	Cell Signaling Technology # 9741
Monoclonal rabbit-total-eIF4E	Cell Signaling Technology # 2067
Monoclonal rabbit-anti-phospho-S6 Ribosomal Protein	Cell Signaling Technology # 4858
Monoclonal rabbit-anti-total-S6 Ribosomal Protein	Cell Signaling Technology # 2217
Monoclonal rabbit-anti-TAB1	Cell Signaling Technology # 3226
Monoclonal rabbit-anti-phospho-Smad1/5/9	Cell Signaling Technology # 13820
Monoclonal rabbit-anti-total-Smad1	Cell Signaling Technology # 6944
Monoclonal rabbit-anti-phospho-Smad2	Cell Signaling Technology # 3108
Monoclonal rabbit-anti-total-Smad2	Cell Signaling Technology # 5339
Polyclonal rabbit-anti-Laminin	Sigma # L9393
Monoclonal mouse-anti-Pax7	DSHB Cat# pax7
Monoclonal mouse-anti-eMyHC	DSHB Cat# F1.652
Monoclonal mouse-anti-F4/80 (BM8)	Invitrogen # 45-4801-82
Polyclonal goat-anti-rabbit IgG Alexa Fluor 568	Invitrogen # A-11036
Polyclonal goat-anti-mouse IgG Alexa Fluor 568	Invitrogen # A-11004
Polyclonal goat-anti-mouse IgG Alexa Fluor 488	Invitrogen # A32731
Polyclonal goat-anti-mouse IgG Alexa Fluor 555	Invitrogen # A-21127
Polyclonal goat-anti-rabbit IgG Alexa Fluor 488	Invitrogen # A-11034
Polyclonal goat-anti-rabbit IgG Alexa Fluor 647	Invitrogen # A-32733

Table S2. Sequence of the primers used for QRT-PCR in the study.

**Gene Name**	**Forward primer (5'-3')**	**Reverse primer (5'-3')**
Hdac4	CAGATGGACTTTCTGGCCG	CTTGAGCTGCTGCAGCTTC
Dach2	ACTGAAAGTGGCTTTGGATAA	TTCAGACGCTTTTGCATTGTA
Myogenin	CATCCAGTACATTGAGCGCCTA	GAGCAAATGATCTCCTGGGTTG
MAFbx	GTCGCAGCCAAGAAGAGAAAGA	TGCTATCAGCTCCAACAGCCTT
MuRF1	TACTGCATCTCCATGCTGGTG	TGGCGTAGAGGGTGTCAAACTT
MUSA1	TCGTGGAATGGTAATCTTGC	CCTCCCGTTTCTCTATCACG
Atg5	ATCAGACCACGACGGAGCGG	GGCGACTGCGGAAGGACAGA
Atg12	ACAAAGAAATGGGCTGTGGAGC	GCAGTAATGCAGGACCAGTTTACC
Beclin-1	TGAAATCAATGCTGCCTGGG	CCAGAACAGTATAACGGCAACTCC
LC3B	CTGGTGAATGGGCACAGCATG	CGTCCGCTGGTAACATCCCTT
F4/80	CGTCAGGTACGGGATGAATATAAG	CTATGCCATCCACTTCCAAGAT
CD206	GGAATCAAGGGCACAGAGTTA	ATTGTGGAGCAGATGGAA
CD11c	GTGCCCATCAGTTCCTTACA	GAGAAGAACTGTGGAGCTGAC
Bmpr1a	GAAAGACCTGATTGACCAGTCC	CCCATCCATACTTCTCCATAGC
Bmpr1b	GAATACCAGCTTCCCTATCACG	TCTGCCTGAGACACTCATCACT
BMPR2	TTGGACTCATCTACTGGGAGGT	TGGACACAAGAACCTGCATATC
Acvr1b	AAGCTGAGAGTTGGGAGAAG	GGGCTTTAGACTTGGTCTGT
Acvr1c	AACTTTGCCAACAGCTAGTC	GGCAGAGAAGAATGTACACC
TGFβR1	AGTCCTTTAACCCTGTTGGT	AACCTGTGAAGAGAGAAGCA
Acvr1	CCCAACTCTGAAACGGACAT	GTGGTGTTGCATGGGTAATG
BMP7	AGCTTCGTCAACCTAGTGGAAC	CTGGAGCACCTGATAGACTGTG
BMP8a	CCTATTACTGTGAGGGGGAGTG	TGACATTGTTGCTGCTGTCATA
BMP8b	TCCACTTTGACCTAACCCAGAT	GTCAGACTCCCTGTTGGAGTG
BMP13	AAGACTTACTCCATTGCCGAGA	TCGTCCAGTCCTCTGTCTACAA
TGFβ1	CTGAACCAAGGAGACGGAATAC	GGGCTGATCCCGTTGATTT
TGFβ2	GGCTTTCATTTGGCTTGAGATG	CTTCGGGTGAGACCACAAATAG
TGFβ3	GTACATCTGCTCTAGGGAATTGG	CCAGGCAGTGCAAGATATGA
Col1a1	TCAAGATGGTCGCCCTGGAC	CCTTTCCAGGTTCTCCAGCG
Col3a1	GTGAACGTGGCTCTAATGGCAT	AATAGGACCTGGATGCCCACTT
β-actin	CAGGCATTGCTGACAGGATG	TGCTGATCCACATCTGCTGG
